# Surprising Homolytic Gas Phase Co−C Bond Dissociation Energies of Organometallic Aryl‐Cobinamides Reveal Notable Non‐Bonded Intramolecular Interactions

**DOI:** 10.1002/chem.202004589

**Published:** 2021-03-25

**Authors:** Alexandra Tsybizova, Christopher Brenig, Christoph Kieninger, Bernhard Kräutler, Peter Chen

**Affiliations:** ^1^ Laboratorium für Organische Chemie Department of Chemistry and Applied Biosciences ETH Zürich Zürich Switzerland; ^2^ Institute of Organic Chemistry & Center of Molecular Biosciences University of Innsbruck Innsbruck Austria

**Keywords:** bond dissociation energy, cobinamide, mass spectrometry, vitamins

## Abstract

Aryl‐cobalamins are a new class of organometallic structural mimics of vitamin B_12_ designed as potential ‘antivitamins B_12_’. Here, the first cationic aryl‐cobinamides are described, which were synthesized using the newly developed diaryl‐iodonium method. The aryl‐cobinamides were obtained as pairs of organometallic coordination isomers, the stereo‐structure of which was unambiguously assigned based on homo‐ and heteronuclear NMR spectra. The availability of isomers with axial attachment of the aryl group, either at the ‘beta’ or at the ‘alpha’ face of the cobalt‐center allowed for an unprecedented comparison of the organometallic reactivity of such pairs. The homolytic gas‐phase bond dissociation energies (BDEs) of the coordination‐isomeric phenyl‐ and 4‐ethylphenyl‐cobinamides were determined by ESI‐MS threshold CID experiments, furnishing (Co−C${}_{\mathrm{sp}{}^{2}}$ )‐BDEs of 38.4 and 40.6 kcal mol^−1^, respectively, for the two β‐isomers, and the larger BDEs of 46.6 and 43.8 kcal mol^−1^ for the corresponding α‐isomers. Surprisingly, the observed (Co−C${}_{\mathrm{sp}{}^{2}}$ )‐BDEs of the Co_β_‐aryl‐cobinamides were smaller than the (Co−C${}_{\mathrm{sp}{}^{3}}$ )‐BDE of Co_β_‐methyl‐cobinamide. DFT studies and the magnitudes of the experimental (Co−C${}_{\mathrm{sp}{}^{2}}$ )‐BDEs revealed relevant contributions of non‐bonded interactions in aryl‐cobinamides, notably steric strain between the aryl and the cobalt‐corrin moieties and non‐bonded interactions with and among the peripheral sidechains.

## Graphical abstract

**The crucial resistance** of aryl‐cobalamins, as antivitamins B_12_, against metabolic conversion into B_12_ cofactors has been traced to their presumed strong organometallic bond. The strength of the Co−C bond of aryl‐cobinamides was examined here by ESI‐MS threshold collision‐induced dissociation (CID) experiments. Surprisingly, the Co−C${}_{\mathrm{sp}{}^{2}}$ bonds of these aryl‐corrins were less strong than the Co−C${}_{\mathrm{sp}{}^{3}}$ bond in their methyl analogue, revealing the relevance of non‐bonded interactions.

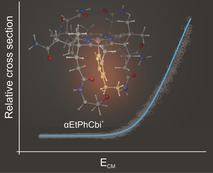

## Introduction

The cobalt‐corrins have held an exceptional place in the sciences,^[1]^ ever since the red vitamin B_12_ was isolated as the enigmatic ‘extrinsic anti‐pernicious anemia factor’.^[2]^ When coenzyme B_12_ (adenosylcobalamin, AdoCbl)^[3]^ was characterized by crystallography in the early 1960s,^[4]^ the critical contribution of organometallic chemistry to life processes was revealed.[^5^, ^6^] The involvement of AdoCbl and of its organometallic analogue methylcobalamin (MeCbl) in intriguing natural processes, indicate the importance of the chemistry of the Co−C bond of the natural corrinoids.[^1h^, ^1j^, ^5^, ^6^, ^7^] Hence, the organometallic reactivity of AdoCbl and of MeCbl has been the specific subject of intense research efforts.[^1e^, ^1h^, ^8^] The nature of AdoCbl as a ‘reversibly functioning’ source of the 5’‐adenosyl radical has been identified as the basis for its role as organometallic coenzyme.^[5]^ However, the molecular mechanics of the enzyme‐controlled initiation of the AdoCbl‐dependent enzymatic radical reactions has remained puzzling, which involves a substrate‐induced activation of AdoCbl towards homolysis of its Co−C bond.[^1e^, ^7^, ^9^]

**Scheme 1:**
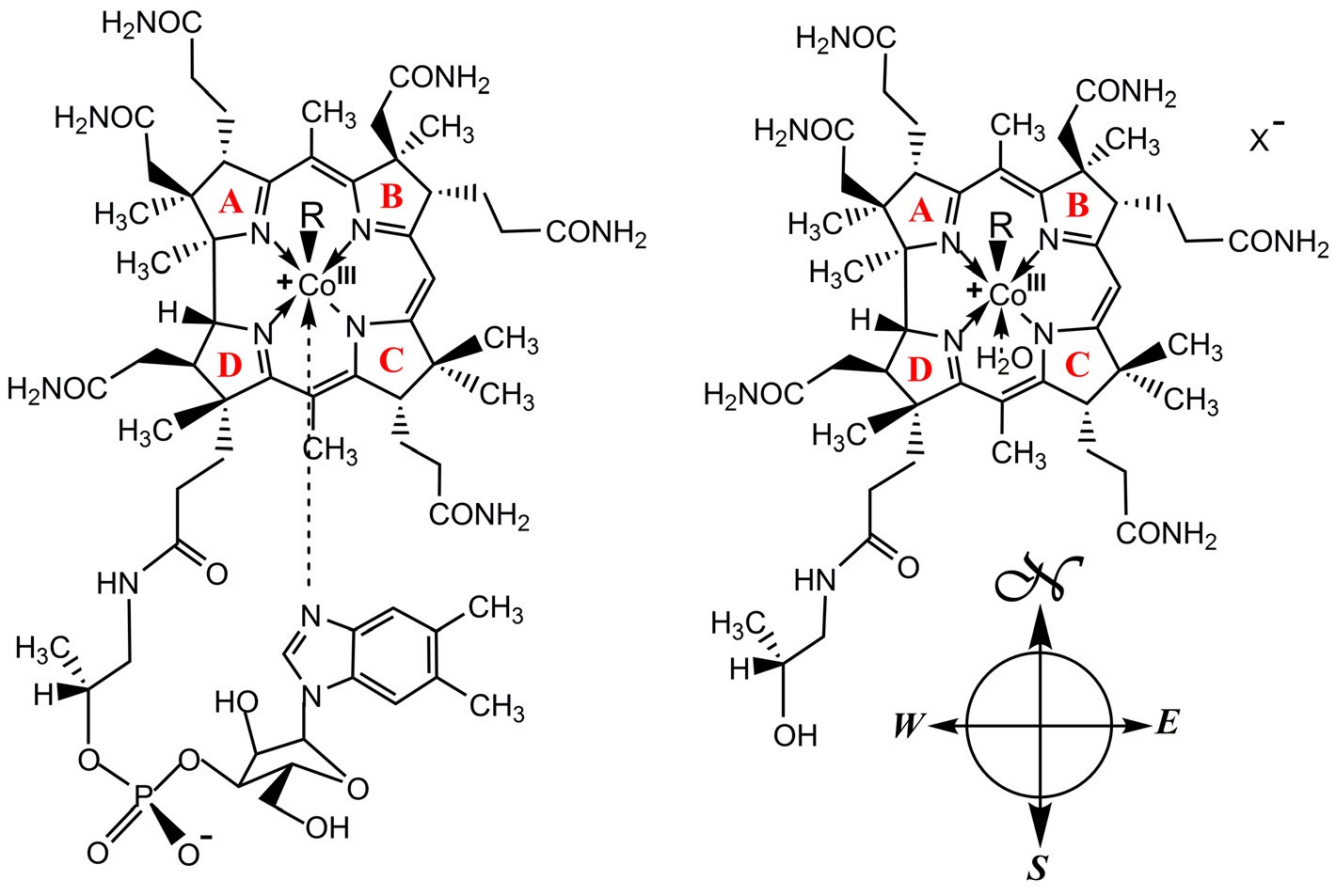
Structural formulae of vitamin B_12_ derivatives. Left. Cobalamins: vitamin B_12_ (CNCbl, R=CN), coenzyme B_12_ (AdoCbl, *R*=5’‐deoxyadenosyl), methylcobalamin (MeCbl, R=CH_3_), 4‐etyhlphenylcobalamin (EtPhCbl, *R*=4‐ethylphenyl). Right. Organometallic cobinamides (various counter ions X^−^; X=Cl in ref. [17]): Co_β_‐5’‐adenosyl‐cobinamide (AdoCbi, *R*=5’‐deoxyadenosyl) and Co_β_‐methyl‐cobinamide (MeCbi, R=CH_3_) and indication of the four directions in the molecular compass (some of which were used in the present text).

Thus, the crucial strengths of the Co−C bonds of AdoCbl and of MeCbl have been the object of a variety of experimental[^5^, ^10^] and theoretical studies.^[11]^ Experimental homolytic Co−C bond dissociation energies (BDEs) of AdoCbl have, so far, been derived by kinetic methods, affording 31.4±1.5^[10]^ kcal mol^−1^ in ethylene glycol and 30±2 kcal mol^−1[12]^ in aqueous solution. The Co−C BDE of MeCbl has also been studied in ethylene glycol by the kinetic approach, furnishing a value of 37±3 kcal mol^−1^.^[13]^ Photoacoustic calorimetry (PAC) with MeCbl in water indicated a Co−C BDE of 36±4 kcal mol^−1[14]^ The Co−C BDE of Co_β_‐adenosyl‐cobinamide (AdoCbi), the ‘incomplete’ analogue of AdoCbl (Scheme 1), was likewise determined by kinetic analysis in aqueous solution as 34.5±1.8 kcal mol^−1^.^[9a]^ For Co_β_‐methyl‐cobinamide (MeCbi) PAC provided a Co‐C DBE of 37±4 kcal mol^−1^.^[14]^ The overlapping values for the Co−C BDE of the ‘complete corrinoid’ MeCbl and of the ‘incomplete’ MeCbi indicated an insignificant effect of the coordination of the 5,6‐dimethylbenzimidazole (DMB) moiety in the Cbls, consistent with thermodynamic studies that indicated a small stabilization by Δ*H*_o_=−2 kcal mol^−1^ in aqueous solution.^[15]^ Cage effects were large in the thermolysis of AdoCbi in aqueous solution,^[16]^ reducing the observed effective homolysis rate and therefore increasing the apparent BDE value. In the gas‐phase homolytic Co−C bond dissociation energies of the cations AdoCbi^+^ (41.5±1.2 kcal mol^−1^) and MeCbi^+^ (44.6±0.8 kcal mol^−1^) were measured by electrospray ionization mass spectrometry (ESI‐MS),^[17]^ utilizing a special fitting of energy‐resolved threshold collision‐induced dissociation (CID) cross‐sections, developed by the Chen group.^[18]^ This approach allowed for benchmarking of inherent electronic structure effects separate from often ill‐defined, and difficult‐to‐model, solvent effects. Unfortunately, significant discrepancies between the derived experimental values and computationally predicted quantitative data continue to prevail, with active investigations of the methodological approximations underlying the experiment, on the one hand, and those underlying the computational approaches, on the other, continuing.[^11d^, ^19^] Hence, important factors that contribute to the Co−C bond strengths in B_12_‐cofactors have largely remained poorly quantified, hampering further insights into the reactivity of the B_12_‐cofactors.

Recently, aryl‐cobalamins were first described,^[20]^ designed as ‘antivitamins B_12_’, which are structural vitamin B_12_ mimics featuring complete resistance against Co−C bond cleavage[^20a^, ^21^] by the B_12_‐tinkerer enzyme CblC.^[22]^ Indeed, the original version of these ‘antivitamins B_12_’, the aryl‐cobalamin 4‐ethylphenyl‐Cbl (EtPhCbl),^[20a]^ induced functional B_12_‐deficiency in laboratory mice,^[23]^ resulted in a remarkable antibacterial effect,^[24]^ and it also underwent photo‐induced homolytic cleavage of its Co−C bond with a remarkably low quantum yield.^[25]^ In order to gain experimental insights into the main factors responsible for the resistance of EtPhCbl against cleavage of its Co−C${}_{\mathrm{sp}{}^{2}}$ bond, we were interested in a study of the Co−C BDEs of suitable aryl‐corrins. We report here the synthesis of cationic aryl‐cobinamides, obtained as unprecedented pairs of Co_α_‐ and Co_β_‐coordination isomers, and the analysis of their homolytic Co−C BDEs in the gas phase by combined ESI‐MS and CID experiments.^[17]^ From a comparison of the newly‐determined BDEs for the aryl‐cobinamides to the previously determined BDEs for methyl‐cobinamide, we deduce that the antivitamin activity of the aryl‐cobalamins likely derives from stereoelectronic rather than thermochemical considerations. Indeed, unexpectedly low BDEs were observed for the aryl‐cobinamides, which are proposed to be weakened by steric interactions between their aryl‐ and corrin moieties. Furthermore, the difference in BDE between the coordination isomers points to an important and surprisingly large contribution to the BDE by noncovalent interactions involving the sidechains along the periphery of the corrin. These factors, in turn, provide potentially relevant mechanisms for the modulation of the crucial strength of the Co−C bond of the natural corrins in the radical generation activity in the enzymes, as well as in solution.

## Experimental Section

### Threshold CID measurements

Electrospray ionization mass spectrometry (ESI‐MS) was used as a primary research technique for bringing the ions from solution to the gas phase. Methanol solutions (5 μm) of the respective cobinamides (Cbis) were sprayed and their respective cationic complexes RCbi^+^ were observed. Qualitative collision‐induced dissociation confirmed the presence of only one dissociation channel, which corresponded to the Co−C bond cleavage.

Threshold collision induced dissociation (T‐CID)^[26]^ experiments allow for determination of bond strengths in the investigated complexes if certain conditions are met. Briefly, during T‐CID the ions of interest collide with noble gas atoms and undergo fragmentation. The intensities of the parent and fragment ions are monitored as a function of collision energy. Statistical thermodynamics is then used to model the experiment and deconvolute the bond dissociation energy from the experimental data. This algorithm (called l‐CID for ligand collision‐induced dissociation) was recently developed in Chen group (see https://chen.ethz.ch/journal‐articles/software.html) and since then is being used extensively for the determination of accurate thermochemical data.^[18]^

T‐CID measurements were performed on the Thermo Scientific TSQ Quantum Ultra tandem mass spectrometer, modified similarly to the Finnigan MAT TSQ‐700 spectrometer used for these measurements in earlier reports.^[27]^

### Quantum chemical calculations

Density functional theory (DFT) calculations were performed with the Gaussian 09 suite.^[28]^ For the computational studies, initial structures of the cobinamides, as well as their radical fragments were obtained with the CREST program (version 2.7.1),^[29]^ which typically produced approximately 200 conformers spanning a range of about 6 kcal mol^−1^. The ten best structures for each of the species, identified in the CREST conformational search, spanned a narrower range of energies, typically less than 2 kcal mol^−1^. These ten structures for each species were then re‐optimized with the BP86 functional, (which is recommended for geometry and Co−C bond dissociation calculations of alkylcorrinoids by a number of benchmark studies)[^11c^, ^30^] in combination with the def2‐TZVP basis set. Density fitting was used to improve the performance of BP86 functional; the Weigend06 (W06) density fitting basis set^[31]^ was used. Dispersion effects were accommodated by Grimme's D3 dispersion correction. Geometry optimization was performed both with and without the D3 correction, for comparison. The Results section reports the BP86‐D3/def2‐TZVP//BP86‐D3/def2‐TZVP structures and energies. Upon re‐optimization, the range of energies found for each ensemble of conformations spanned up to 8 kcal mol^−1^. The final minimum energy structures, when inspected manually, looked reasonable with what could be interpreted as maximized stabilizing interactions. Frequency analyses were performed to confirm the nature of located stationary points as true minima with no imaginary frequencies, and to provide zero‐point energy corrections.

## Results

### Synthesis and spectroscopy

Four organometallic aryl‐cobinamides were prepared from dicyanocobinamide^[32]^ by applying the recently developed methodology for Co‐arylation of Cbls using diaryliodonium salts^[20b]^ and isolated as the pairwise coordination isomeric tetrafluoroborates (Co_α_‐PhCbi[BF_4_]=αPhCbi, Co_β_‐PhCbi[BF_4_]=βPhCbi, Co_α_‐EtPhCbi[BF_4_]=αEtPhCbi and Co_β_‐EtPhCbi[BF_4_]=βEtPhCbi) (see Supporting Information). In brief, aquocyano‐cobinamide (H_2_OCNCbi) was first reduced with NaBH_4_ under argon atmosphere and subsequently treated with diaryliodonium salts at room temperature (i.e. diphenyliodonium chloride or di(4‐ethylphenyl) iodonium tetrafluoroborate),^[33]^ furnishing a pairwise mixture of α‐ and β‐coordination isomeric organometallic aryl‐cobinamides PhCbi or EtPhCbi. The isomeric products were separated by semi‐automated reversed phase MPLC. Orange Co_α_‐aryl‐cobinamides (αPhCbi or αEtPhCbi) eluted first in both preparations at approx. 15 % MeOH, followed by the more apolar yellow Co_β_‐aryl‐cobinamides (βPhCbi or βEtPhCbi). In a subsequent step, the isolated aryl‐Cbi samples were loaded on RP‐18 cartridges and anion exchange was performed by washing with 0.1 m NaBF_4_ solution. The products were obtained as the tetrafluoroborate salts αPhCbi (58 %) and βPhCbi (24 %), as well as αEtPhCbi (62 %) and βEtPhCbi (21 %) and precipitated to furnish the solid aryl‐cobinamides in a pairwise combined yield of 82 to 83 %.

**Figure 1:**
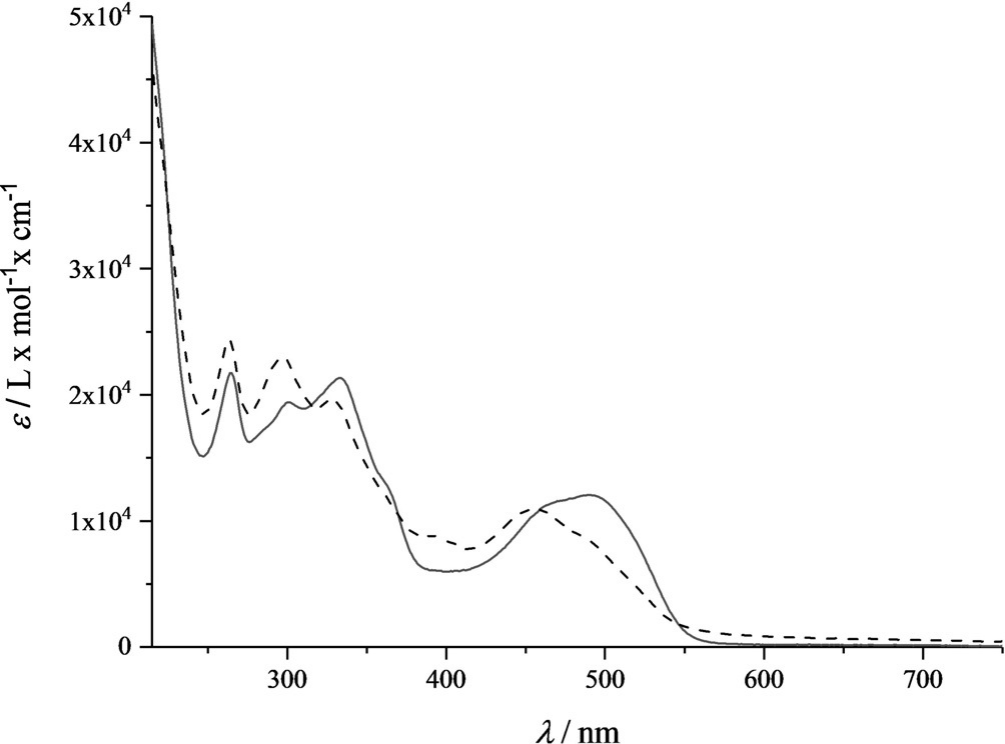
UV/Vis‐spectra of coordination isomeric phenyl‐cobinamide tetrafluoroborates (*c*=0.043 mm in H_2_O). Full line: Co_α_‐isomer αPhCbi, dashed line: Co_β_‐isomer βPhCbi.

The novel organometallic aryl‐cobinamides were isolated as pure powders and characterized via their UV/Vis, homo‐ and heteronuclear 2D NMR and their ESI‐MS spectra. The UV/Vis spectrum of βPhCbi in aqueous solution features absorption bands at 484 and 453 nm (α‐ and β‐bands), 328 nm (γ‐band), 296 and 264 nm (Figure 1). It is similar to the absorption spectra of the DMB‐protonated (‘base‐off’) form of Co_β_‐PhCbl (=PhCbl)^[20b]^ and of the ‘organometallic Co_β_‐alkyl‐cobinamides, such as MeCbi,^[17]^ but shifted hypsochromically compared to the spectrum of (DMB‐coordinated ‘base‐on’) PhCbl.^[20b]^ The diastereomeric counterpart α PhCbi features its αβ‐bands slightly shifted to longer wavelengths at 490 nm and 465 nm. Thus, the UV/Vis‐spectra of coordination isomeric pairs βPhCbi and αPhCbi (Figure 1) exhibit similar differences as corresponding pairs of coordination isomers of ‘incomplete’ alkylcorrins.^[34]^ As expected, the UV/Vis‐spectra of the pairs βPhCbi and βEtPhCbi, or αPhCbi and αEtPhCbi are virtually superimposable, pairwise.

ESI‐MS of the four aryl‐cobinamides α/βPhCbi and α/βEtPhCbi showed single dominating positive ions at *m*/*z* 1066.5 and 1094.5, respectively (see Supporting Information), reflecting the intact sum formulas of the respective cobinamide cations C_54_H_77_CoN_11_O_8_^+^ and C_56_H_81_CoN_11_O_8_^+^. Loss of the aryl groups was not significant under the mild conditions used for these ESI‐MS analyses.

**Figure 2:**
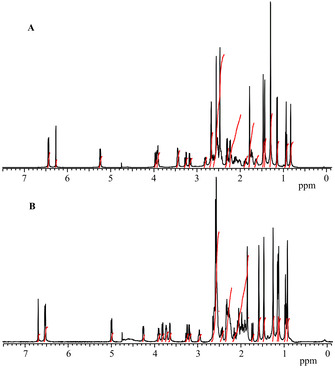
500 MHz ^1^H NMR‐spectra of coordination isomeric 4‐ethylphenylcobinamide tetrafluoroborates (*c*=8.3 mm, in D_2_O, 298 K). A: αEtPhCbi, B: βEtPhCbi.

^1^H,^1^H‐homonuclear and ^1^H,^13^C‐heteronuclear NMR spectra of the newly prepared aryl‐cobinamides allowed for the unambiguous assignment of their structures. In the low field region of a 500 MHz ^1^H NMR spectrum of αEtPhCbi in D_2_O (see Figure 2) the two doublets of the *ortho* and *meta* protons of the phenyl moiety were found at 5.24 and 6.44 ppm, respectively. Corresponding doublets occurred at 5.00 and 6.53 ppm, respectively, in the spectrum of βEtPhCbi. One doublet and two triplets were, likewise, seen in the low field for the aromatic protons of the Co‐bound phenyl group in the spectrum of αPhCbi at 5.36, 6.55 and 6.68 ppm, respectively, with the doublet of the two *ortho* protons shifted strongest towards high field. In the case of the diastereomeric βPhCbi (see Supporting Information), the signals of the *ortho*, *meta* and *para* protons occurred at 5.10 ppm, 6.62 ppm and 6.75 ppm. The vinyl protons HC10 gave rise to singlets at low field as well, that is, at 6.24 ppm or at 6.27 ppm for the α‐aryl‐cobinamides αPhCbi and αEtPhCbi, and at 6.66 and at 6.69 ppm for the corresponding β‐aryl‐cobinamides βPhCbi and βEtPhCbi. The ^1^H NMR spectra of αEtPhCbi and βEtPhCbi revealed only slight differences in chemical shifts of large parts of the assigned protons. The phenyl‐bound ethyl group produced an additional triplet at 0.93 ppm and a quartet at 2.30 ppm in the spectrum of βEtPhCbi (or, correspondingly, at 0.97 and around 2.25 ppm for αEtPhCbi). The signals of the aromatic protons of the ethyl‐substituted or unsubstituted phenyl units exhibited clear diagnostic differences. The isotropic nature of the pairs of signals of HC2L and HC6L, as well as of HC3L and HC5L, indicated an effectively identical environment, as is generated by rapid rotation of the phenyl substituent around its Co−C bond. All 54 C‐atoms of the PhCbis were assigned via clear ^1^H,^13^C‐heteronuclear correlations in HMBC and HSQC spectra (see Supporting Information).^11^B and ^19^F NMR spectra of aqueous 1.84 mm solutions of αPhCbi and βPhCbi confirmed the presence of tetrafluoroborate as non‐coordinated and effectively symmetrical, solvated counterion (see Supporting Information).

Characteristic and similar high field shifts of the methyl group singlets C1A to 0.78 and 0.83 ppm were noted in the spectra of αPhCbi and αEtPhCbi, respectively, whereas the corresponding signals occurred as broad singlets at 1.1–1.15 ppm and 1.12 ppm for the β‐aryl‐cobinamides βPhCbi and βEtPhCbi, respectively. The high field shifts of the methyl group signals H_3_C1A by about 0.4 ppm in the α‐isomers reflect the shielding effect by the nearby coordinated phenyl group at the lower (α‐) face of the corrin macrocycle, for atom numbering see Supporting Information). Similar anisotropic effects of the organometallic phenyl group were noted with the ‘base‐off’ αPhCbl.^[20b]^

**Figure 3:**
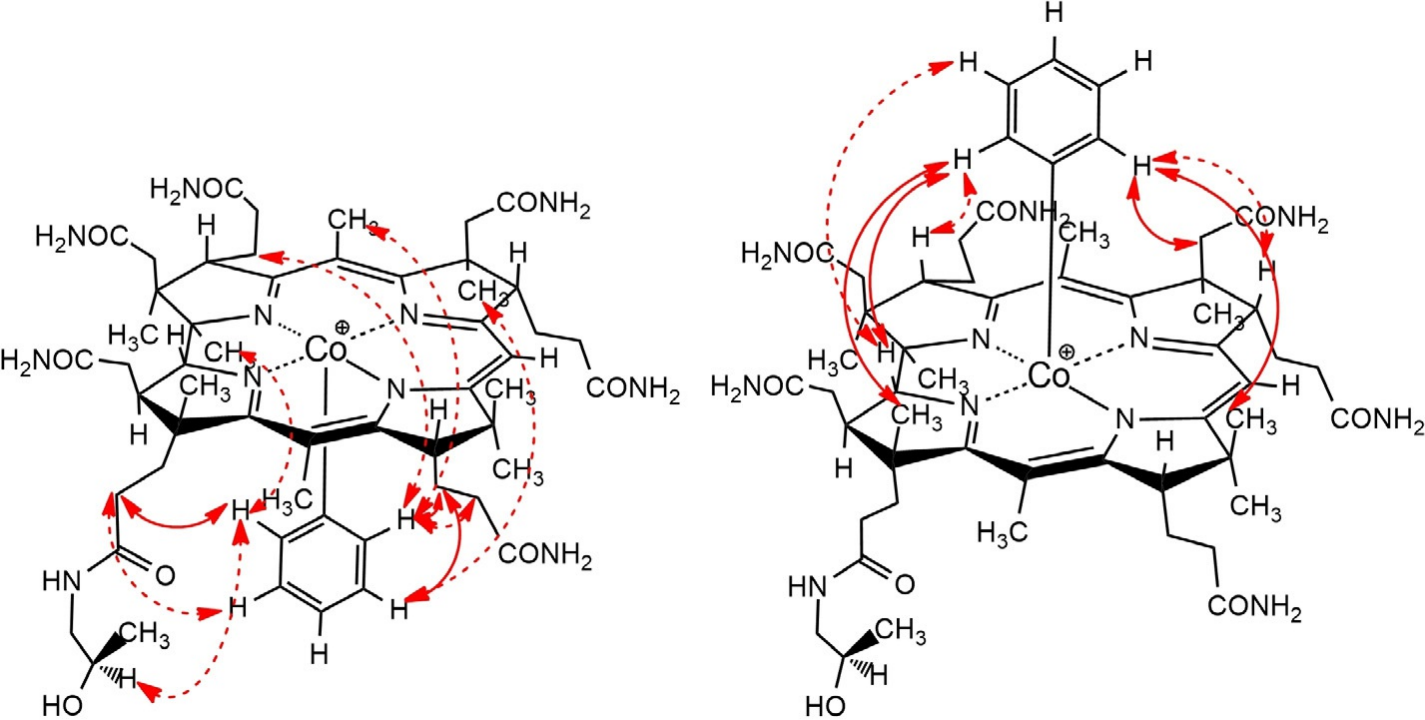
Assignment of the axial position of the phenyl group in (left) αPhCbi and (right) βPhCbi from ^1^H,^1^H‐NOE correlations between protons of the aryl group and protons located on the corrin ligand. The NOE correlations derived from ^1^H,^1^H ROESY experiments also assisted in the here presented signal assignments in the ^1^H NMR spectra (note: pairs of *ortho* and *meta*‐protons had identical chemical shift). Solid line: strong correlation, dashed line: weak to medium correlation.

**Scheme 2:**
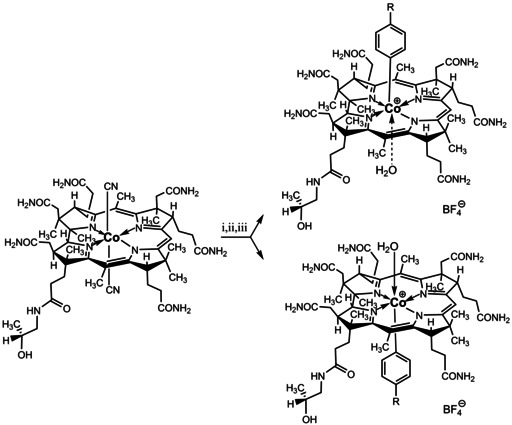
Outline of the synthesis and structural formulae (right) of Co_β_‐aryl‐cobinamides (top) and Co_α_‐aryl‐cobinamides (bottom) as tetrafluoroborate salts, respectively (R=H: αPhCbi and βPhCbi; R=ethyl: αEtPhCbi and βEtPhCbi). The aryl‐cobinamides are depicted here as ion pairs with a(n experimentally not verified) *trans*‐axial water ligand; i) aqueous HOAc, room temperature, raw isolation; ii) sodium borohydride in water, room temperature, under argon; iii) diphenyliodonium chloride or di(4‐ethylphenyl)‐iodonium tetrafluoroborate, room temperature, under argon, workup using sodium tetrafluoroborate (see text and Supporting Information for details).

The axial site of attachment of the organometallic group of the non‐crystalline Co_α_‐ and Co_β_‐aryl‐cobinamide tetrafluoroborates was established unambiguously with further homo‐ and heteronuclear NMR studies. ^1^H,^1^H ROESY enabled detection of strong NOE correlations between the *ortho* and *meta* protons of the phenyl group of αPhCbi and methylene groups of the propionamide side chains C131, C132 and C171, as well as weak NOEs to the methyl groups C1A, C7A and C51, confirming the coordination of the phenyl group on the lower (α‐) side in αEtPhCbi and αPhCbi (Figure 3). As expected, complementary effects were found in the spectra of βPhCbi and βEtPhCbi. In these β‐isomers, strong NOE correlations between the *ortho* protons of the phenyl groups and the protons of the methyl groups at C12B and C17B, as well as the corrin protons HC19 and HC3, allowing an unambiguous location of their phenyl ligands at the β‐axial position. The observed NOE correlations between the aryl group and the corrin ligand are consistent with a predominant north‐south orientation of the organometallic moiety, as was similarly seen in the crystal structures of the corresponding cobalamins EtPhCbl^[20a]^ and PhCbl.^[20b]^ Coordination of a water molecule on the free axial position of the aryl‐cobinamides (as depicted in Scheme 2) would complete a hexa‐coordinate ligand shell, as is typical of Co^III^ centers, but good experimental evidence for the coordination of axial aquo‐ligands in organometallic cobinamides and in other ‘incomplete organometallic corrinoids’ in aqueous solution is lacking.^[35]^ QM/MM‐calculations indicate a stable 6‐coordinate Co_α_‐aquo complex of the methylcobinamide cation (MeCbi^+^).^[36]^

Preliminary qualitative photolysis studies revealed a light‐induced cleavage of the Co−C bond, when an aerated aqueous solution of αEtPhCbi was irradiated with diffuse light (Hg fluorescence tube) for 78 h. HPLC analyses of the photolysis mixture showed photolytic degradation of αEtPhCbi and 37 % isomerization to the corresponding β‐aryl‐cobinamide, βEtPhCbi. The isomerization provides evidence for a light‐induced homolytic Co−C bond cleavage of αEtPhCbi and subsequent “trapping” of the EtPh‐radicals by the concomitantly formed cob(II)inamide intermediate. Under the same conditions (i.e., 78 h of irradiation) βEtPhCbi showed only insignificant degradation or isomerization. Indeed, the photohomolysis of the Co_β_−C bond of EtPhCbl has been classified as a low quantum yield reaction.^[25]^

### Co−C BDE measurements

Experimental bond dissociation energies for the α‐ and β‐diastereomeric cations of phenylcobinamide (PhCbi^+^) and 4‐ethylphenyl‐cobinamide (EtPhCbi^+^) were measured by threshold collision‐induced dissociation (T‐CID) in a modified ESI‐MS/MS spectrometer. A plot of the energy‐resolved reactive cross‐section for carefully thermalized ions, extrapolated to single‐collision conditions, and fitted with the l‐CID program, produces the bond dissociation energies, BDE, listed in Table 1.

**Table 1:** Experimental gas**‐**phase BDEs (kcal mol^−1^).

Aryl‐cobinamide cation	BDE
βEtPhCbi^+^	40.6±1.2
βPhCbi^+^	38.4±0.8
αEtPhCbi^+^	43.8±0.9
αPhCbi^+^	46.6±1.5

**Figure 4:**
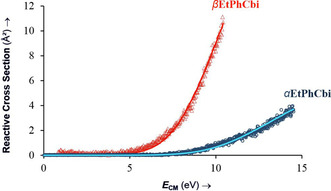
Representative examples of the zero‐pressure‐extrapolated cross sections (circles and triangles) with L‐CID‐fitted curves (lines) for α‐ and βEtPhCbi, respectively.

Representative T‐CID curves for α‐ and β‐EtPhCbi from two separate measurements are shown, superimposed in the Figure 4 below, from which the quality of the data and the quality of the fits may be seen. While the l‐CID program extracts *E*_0_, which in the case of a homolytic dissociation corresponds to the bond dissociation energy, one can surmise from the curves themselves, even without an explicit fitting, that the α‐ and β‐diastereomers have significantly different BDEs, with the β‐BDE being lower. In practice, the T‐CID curves are very sensitive to the BDE, with differences of much less than 1 kcal mol^−1^ shifting a curve perceptibly if all other parameters were to be held constant. The extracted difference of 3.2 kcal mol^−1^ produces the distinctly different T‐CID curves in Figure 4 for the dissociation of diastereomeric EtPhCbi^+^ molecular ions that differ only by the face to which the aryl group is attached. The difference for the diastereomeric PhCbi^+^ ions, 8.2 kcal mol^−1^, is even larger (see Supporting Information).

### DFT‐calculations of Co−C BDEs

The calculated BDEs are shown in Table 2. As mentioned above, the ten best conformations found by CREST for each of the α‐ and β‐derivatives, as well as the Cbi^+^ ion produced by Co−C bond scission, were re‐optimized with the BP86‐D3/def2‐TZVP method. We note in passing that both β‐isomers were found to prefer compact conformers, in which the propionamide side chains undergo hydrogen bond interactions among themselves and can stabilize the coordinatively unsaturated 5‐coordinate Co^III^ species (or 4‐coordinate Co^II^ species). This finding is not surprising for the gas‐phase case, considering that the stabilizing intramolecular interactions in the compact conformer would not be balanced out against stabilizing intermolecular interactions with solvent molecules that, for example, more extended conformers, and the cobalt‐center, would have had in aqueous solution.

**Table 2:** Computed BDEs (ZPE corrected) for the most stable conformers at BP86 and BP86‐D3 level of theory with def2‐TZVP basis set in kcal mol^−1^.

Aryl‐cobinamide cation	BP86	BP86‐D3
βEtPhCbi^+^	45.4	68.9
βPhCbi^+^	44.7	67.4
αEtPhCbi^+^	31.4	48.7
αPhCbi^+^	28.6	45.7

**Figure 5:**
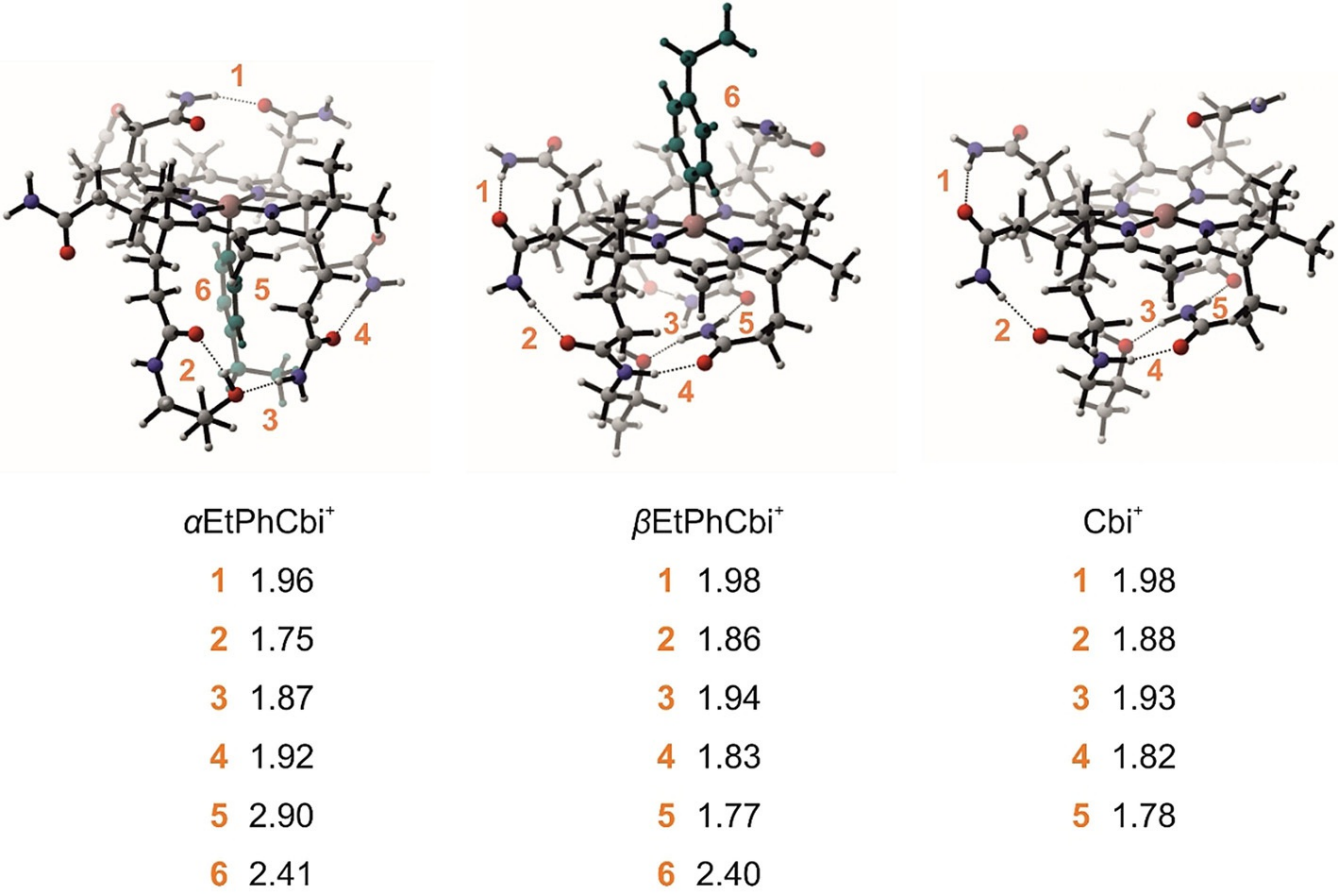
BP86‐D3/def2‐TZVP‐optimized structures for the most stable conformers of EtPhCbi and the Cbi^+^ fragment found by CREST. The atoms in the aryl substituent are colored in green for clarity. Non‐covalent interactions are labeled in orange, together with the distances reported in Å .

In addition to the computed BDEs, we depict the most stable conformers for the αEtPhCbi^+^ βEtPhCbi^+^, and Cbi^+^ fragment, respectively, in Figure 5. The global view in Figure 5 orients the molecules in the ‘North to South’ direction, to highlight the salient non‐bonded contacts among the side‐chains, and between the side‐chains and the aryl moiety.

Table 3 depicts the computed structures of α‐ (left) and βEtPhCbi^+^ (right) in a close‐up view of the immediate vicinity of the central Co atom, emphasizing the various distances to the “best” plane defined according to the four nitrogen atoms in the corrin ring as executed in the Mercury crystallographic software.^[37]^ It shows both of the isomers rotated by 90° around the axis of the Co−C bond, relative to the orientation in Figure 5. In addition, the α‐diastereomer (displayed on the left) is flipped 180° in order to orient the aryl group to the top. The distances in the close‐up view are also given in tabulated form below, where corresponding X‐ray crystallographic distances from MeCbl and two related aryl‐Cbls are also listed, for a comparison.

**Table 3:** Close‐up view of the BP86‐D3/def2‐TZVP‐optimized gas‐phase structures for the most stable conformers of ArCbi^+^ together with the corresponding distances of the *ortho*‐hydrogens on the aryl rings to the best plane through the four ‘inner’ corrin *N*‐atoms found by Mercury crystallographic software, the lengths of the Co−C bonds, and the distances of the Co atoms from the best corrin *N*‐atom plane, all compared to the corresponding distances in the X‐ray crystal structures for EtPhCbl, PhCbl, and MeCbl, as well as a calculated structure of MeCbi^+^.

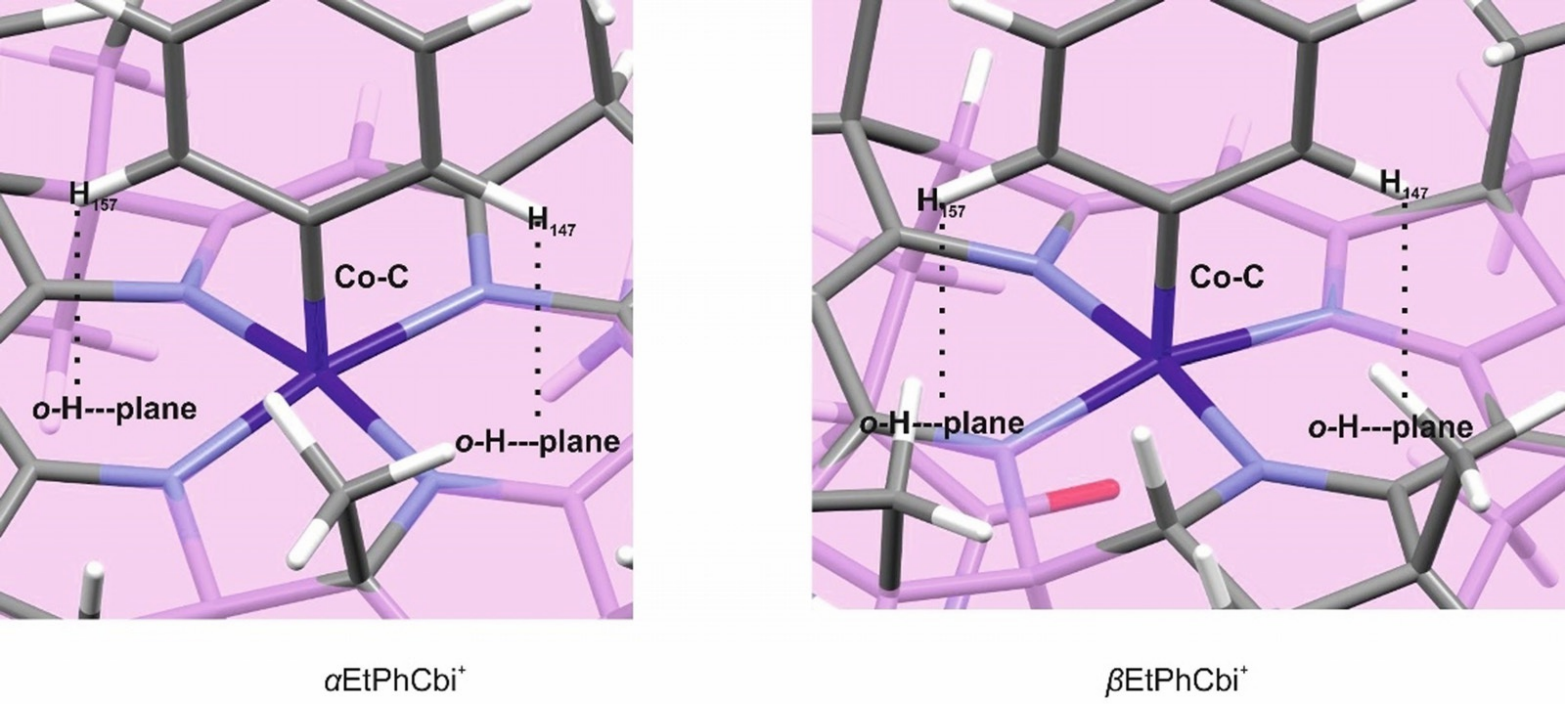				
	Distance [Å]			
Compound	*o‐*H_157_‐plane	*o‐*H_147_‐plane	Co−C	Co‐plane
αEtPhCbi^+^ (gas, calcd)	2.349	2.196	1.934	0.143
βEtPhCbi^+^ (gas, calcd)	2.389	2.195	1.947	0.153
αPhCbi^+^ (gas, calcd)	2.372	2.165	1.933	0.161
βPhCbi^+^ (gas, calcd)	2.382	2.194	1.947	0.155
EtPhCbl (crystal, exptl)^[20a]^	2.397	2.154	1.981	0.029
PhCbl (crystal, exptl)^[20b]^	2.398	2.190	1.988	0.042
MeCbl (crystal, exptl)^[38]^	NA	NA	1.979	0.020
MeCbi^+^ (gas, calcd)^[36]^	NA	NA	1.970	0.097

Visual inspection of the computed structures in Figure 5 and Table 3 finds several noteworthy points. The calculated lengths of the Co−C${}_{\mathrm{sp}{}^{2}}$ bonds in the ArCbi^+^s (roughly 1.94 Å) are close, but shorter by roughly 0.045 Å than the experimental lengths of the Co−C${}_{\mathrm{sp}{}^{2}}$ bonds (1.981 Å and 1.988 Å) of two ArCbls. They are, moreover, significantly longer than the Co−C${}_{\mathrm{sp}{}^{2}}$ bond in vinyl‐Cbl (from crystallography: 1.911 Å)^[39]^ and unexpectedly close to the lengths of the Co−C${}_{\mathrm{sp}{}^{3}}$ bond in MeCbi^+^ (calculated for its 6‐coordinate aquo‐form: 1.970 Å)^[36]^ and MeCbl (from crystallography: 1.979 Å).^[38]^ A significant out of plane position of the Co^III^‐center is calculated for the ArCbi^+^s, increased by roughly 0.1 Å compared to the corresponding crystallographic distances in the two ArCbls. The two geometric features (Co−C bond lengths, out of plane position of cobalt), computed for the 5‐coordinate ArCbi^+^s, add up to remarkably similar close distances of the phenyl *ortho*‐H‐atoms to the best plane through the four ‘inner’ corrin N‐atoms, as also found in ArCbls^[20]^ with 6‐coordinate Co^III^‐centers. Secondly, in Figure 5, one sees striking differences in the number and kind of non‐covalent interactions, as one compares αEtPhCbi^+^, βEtPhCbi^+^, and their common fragment Cbi^+^. We note that the arrangement of hydrogen bonds for our βArCbi^+^ and Cbi^+^ structures agree broadly with those computed by Grimme and co‐workers for (gas phase) MeCbi^+^.^[11d]^ Dissociation of the Co−C bond in βEtPhCbi^+^ produces essentially no change in the hydrogen‐bond network, especially on the α‐face, which can be seen qualitatively in Figure 5. On the other hand, the constellation of non‐covalent interactions changes drastically upon Co‐C cleavage in αEtPhCbi^+^. A more detailed analysis follows in the Discussion.

Comparison of the calculated bond dissociation energies show the relative ordering of BDEs in α‐ and β‐derivatives to be opposite to that observed in the experiment, a phenomenon for which an explanation is needed.

## Discussion

Recent work of our groups has led to the design and synthesis of aryl‐cobalamins^[20]^ for use as ‘antivitamins B_12_’,^[21]^ as well as to first ESI‐MS CID experiments that use an energy‐resolved threshold collision‐induced dissociation technique for the determination of gas phase Co‐C BDEs of cobalt‐corrins.^[17]^ The capacity of the aryl‐cobalamin, 4‐ethylphenyl‐cobalamin, to act as ‘antivitamin B_12_’ was rationalized by the resistance of this organometallic vitamin B_12_ analogue toward ‘tailoring’ by the enzyme CblC.^[20a]^ An increased inertness of the aryl‐cobalamin against nucleophilic or reductive cleavage of its Co−C bond has been implied. Hence, we have been interested in the question of the strength of the Co‐C${}_{\mathrm{sp}{}^{2}}$ bond in aryl‐corrins, which we had presumed to be intrinsically stronger than the Co‐C${}_{\mathrm{sp}{}^{3}}$ bond of the very readily ‘tailored’ alkyl‐Cbls, such as MeCbl.^[20a]^ The here studied aryl‐cobinamides were made available as salts of ‘cationic’ corrins suitably charged for mass spectrometric determination of their homolytic Co−C bond dissociation energies (BDEs). They were prepared as coordination isomers by the recently developed reductive method using diaryliodonium reagents as source of the aryl groups.^[20b]^ The α‐ or β‐diastereomers were obtained in a roughly 3:1 ratio, respectively, in both of the investigated series. Classic synthetic work with cobalamins indicated the formation of the (thermodynamically more stable) organometallic Co_β_‐isomers with a typical high selectivity.^[40]^ Hence, the preferred formation of Co_α_‐isomers of organometallic cobinamides and other ‘incomplete corrinoids’ is mechanistically intriguing,[^6^, ^7b^, ^34^] and has been rationalized on the basis of radical processes in the formation of the organometallic bond.[^20b^, ^34^] Indeed, for the construction of Co‐aryl bonds a radical mechanism was considered to be particularly advantageous.^[20]^ The ‘reductive’ arylation of cobinamides with diaryliodonium salts was conceived as exploiting a reductive formation of an aryl radical^[41]^ and subsequent efficient trapping by the radicaloid cob(II)inamide (Cbi^II^). The here observed 3:1 stereo‐selectivity could be rationalized by the presence of 5‐coordinate Cbi^II^ with significant preference for Co_β_‐coordination of its axial ligand (H_2_O).^[42]^ Under kinetic control, this would lead to preferential combination on the free α ‐face with the hypothetical aryl radical intermediate and, thus, selective formation of α‐aryl‐cobinamide diastereomers.

In contrast to the prior work on MeCbi^+^ and AdoCbi^+^,^[17]^ for which only the β‐diastereomers were available, the novel aspect of the synthesis, the production and separation of the α‐ and β‐diastereomers, presents a unique opportunity to investigate the thermodynamics of Co−C bond cleavage. The measurements by positive‐ion ESI‐MS and CID experiments of the ‘cationic’ aryl‐Co^III^‐corrin moieties of the four aryl‐cobinamides (α‐ and βPhCbi, α‐ and βEtPhCbi) revealed, first of all, that the homolytic BDEs of the Co−C${}_{\mathrm{sp}{}^{2}}$ bonds of the two Co_β_‐isomer cations (βPhCbi^**+**^ and βEtPhCbi^+^) are unexpectedly less than what was found for the corresponding Co−C${}_{\mathrm{sp}{}^{3}}$ bond of MeCbi^+^,^[17]^ which along with the Co−C${}_{\mathrm{sp}{}^{2}}$ bond lengths coming out unexpectedly long, goes counter to the qualitative notions based simply on hybridization. In consequence of these results, the strength of the Co−C in the aryl‐cobalamin EtPhCbl cannot be considered any longer the crucial factor for resistance of EtPhCbl against its ‘tailoring’ by the enzyme CblC.^[20a]^ Indeed, the fundamental lack of reactivity of this antivitamin B_12_ in a nucleophilic displacement most likely comes about primarily from (i) the classification of such a reaction at an un‐activated phenyl carbon as ‘forbidden’,^[43]^ and (ii) from its stringent stereo‐electronic requirement for a backside attack of the nucleophile^[44]^ on the Co−C bond. With its proper substrates, such as MeCbl, the enzyme CblC assists such a directed attack by the protein bound nucleophilic co‐substrate glutathione. Indeed, in the enzyme, the Co−C bond in aryl‐cobalamins is very effectively protected by the aryl group in the direction of the potential thiolate nucleophile attack. Furthermore, the greatly lower quantum yield of photo‐induced homolysis of EtPhCbl,^[25]^ when compared to MeCbl or AdoCbl, can, likewise, not be directly associated with the strength of the Co−C bond of these organo‐cobalamins in the ground state.^[45]^

In search of specific structural factors that may contribute qualitatively to making the Co−C${}_{\mathrm{sp}{}^{2}}$ bonds of the Co_β_‐isomer cations βPhCbi^+^ and βEtPhCbi^+^ less strong than the corresponding Co−C${}_{\mathrm{sp}{}^{3}}$ bond of MeCbi^+^,^[17]^ destabilizing steric interactions between the phenyl and bulky corrin‐ring moieties had been identified in the crystal structures of EtPhCbl^[20a]^ and of PhCbl.^[20b]^ Such non‐bonding interactions between the phenyl and corrin‐ring sections also appear to be responsible for reducing the fold angle of the corrin ligand to the exceptionally low value of (e.g.) 7.6° in EtPhCbl.^[20a]^ Corresponding interactions, only larger, do seem to be present in the computed structures of the aryl cobinamides, Table 3. In particular, the distances from the *ortho* hydrogens on the aryl moiety to the best plane defined by the four corrin nitrogens, 2.196 and 2.349 Å for αEtPhCbi^+^, and 2.195 and 2.389 Å for βEtPhCbi^+^, resemble the 2.154 and 2.397 Å found by X‐ray crystallography for EtPhCbl, for which, as noted above, an unfavorable steric interaction could be inferred.^[20a]^ The distortion of the Co atom out of the best plane defined by the corrin nitrogens is also significantly larger for the ArCbi^+^ structures than for any of the RCbls^[20a]^ or for (calculated) MeCbi^+^ with a hexacoordinate Co‐center.^[36]^ While the comparison to the RCbl structures is fraught with complications due to the DMB ligand coordinated at the other axial site of Co in the cobalamins, as well as the different array of hydrogen‐bonded interactions, the comparison to MeCbi^+^ speaks strongly and unambiguously for an unfavorable steric interaction between the aryl group, specifically the *ortho*‐hydrogens, and the corrin ring, in α‐ and βArCbi^+^. Indeed, steric effects have been inferred earlier among the factors responsible for weakening the Co−C bond of organocorrins.^[46]^ The structural evidence from the present study would be consistent with the experimental observation of a Co‐C${}_{\mathrm{sp}{}^{2}}$ BDE lower in aryl‐corrins than would have otherwise been expected.

**Scheme 3:**
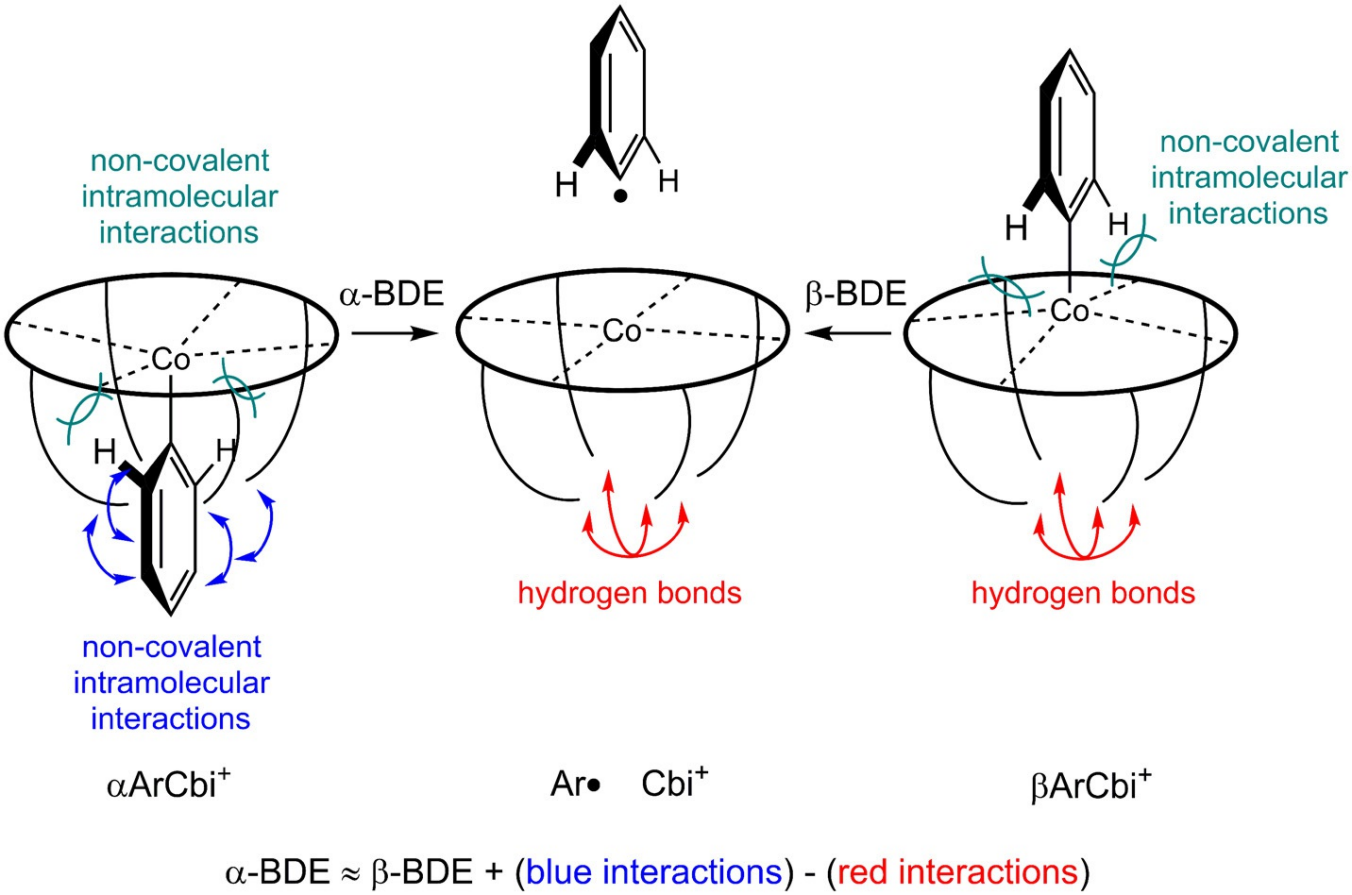
Cartoon with schematically depicted stabilizing non‐covalent interactions in α‐ (blue) and β‐ (red) derivatives, where only the propionamide groups are depicted symbolically. Further non‐covalent interactions, marked in green, affect the absolute magnitude of the BDE but likely do not differentiate α‐ from β‐faces.

An interesting second finding of the gas‐phase MS‐experiments has arisen from the unprecedented determination of the strengths of the Co−C bonds on either face of the corrin‐bound cobalt center, a comparison which could not have been done before, because, for other organo‐corrinoids, both diastereomers were not available. Surprisingly, the Co_α_−C bonds of αPhCbi^+^ and αEtPhCbi^**+**^ were determined to be effectively stronger by 8.2 kcal mol^−1^ or 3.2 kcal mol^−1^ than their respective Co_β_‐C equivalents. In aqueous solution, the Co_β_‐form of MeCbi^+^ is more stable than its Co_α_‐isomer.[^15^, ^40^] The core of the Cbi^+^‐ligand is nearly *C*_2_‐symmetric, with the computed position of the Co^II^‐center in the Cbi^+^ fragment ion lying only 0.016 Å out of the best plane defined by the four corrin nitrogens, this slight perturbation arising from the asymmetry of the substituents of the Cbi‐ligand. One expects, therefore, similar intrinsic reactivity for the two axial coordination sites at the bound cobalt ion in α‐ and β‐ArCbi^+^. Indeed, the difference in the deviation of the Co atom from the plane of the corrin nitrogens, as well as the difference in Co−C bond lengths, in the computed structures, between each αArCbi^+^ and the corresponding β‐diastereomer, amount to only approximately 0.01 Å, as listed in Table 3, which would have suggested similar intrinsic BDEs. Specific structural factors that may make the effective gas‐phase Co−C BDE's of the Co_α_‐isomers (αPhCbi^+^ and αEtPhCbi^+^) larger than those of the two Co_β_‐isomers (βPhCbi^+^ and βEtPhCbi^+^) are, hence, difficult to pin down. However, the remarkable dichotomy of the arrangement of the peripheral groups,^[1d]^ with contributions of the seven amide substituents of the natural corrinoids in particular, should contribute to a different axial reactivity. The four propionamide side chains are oriented downwards (alpha), whereas all three acetamide side chains go ‘up’ (beta). Especially the longer propionamide side chains may undergo significant nonpolar intramolecular interactions with the lipophilic aryl ligands at the lower axial coordination site (blue interactions in Scheme 3) as also found for the DMB‐base in the cobalamins. When situated at an appropriate distance, they also provide a stabilizing interface for a(n external) phenyl group, important for placing an aromatic phenylalanine residue near the crucial empty axial position of the bound 4‐coordinate Co^II^‐cobalamin in the active site of the enzyme adenosyl transferase.^[47]^ In a related way, the present mass spectrometric investigations involve aryl‐corrins with coordinatively unsaturated 5‐coordinate Co^III^‐ and 4‐coordinate Co^II^‐centers, before and after homolytic loss of the aryl group, respectively. Hence, in the gas phase, the propionamide side chains are ‘tucked in’ in the 5‐coordinate aryl Co^III^ β‐corrins and in the 4‐coordinate Co^II^‐corrins, undergoing polar interactions with the coordinatively unsaturated, electrophilic cobalt centers, as seen in the enzyme CblC, to which cobalamins are bound in an activated form featuring a 5‐coordinate Co^III^‐center.^[48]^

To gain deeper structure‐based insights into the experimentally determined BDEs, we performed a number of computational studies. While computed BDEs presented here reproduce neither the trends nor the absolute values for the experimentally determined BDEs, for reasons that we discuss below, they do nevertheless provide important insight into the molecular‐level interactions that likely make the computational treatment of the Co−C BDEs so difficult in this, as well as previous, studies. With a few exceptions (notably for example,[^11c^, ^11d^, ^17^, ^30^, ^36^, ^49^]) previous computational studies of organo‐cobalamins/cobinamides typically truncate the corrin ligand by removing the peripheral substituents bearing polar amide groups,[^11a^, ^11b^, ^11c^, ^19^, ^50^] one of which further bearing an isopropanolamine extension. In our computational study, all of the substituents were retained, and the conformations optimized, because they are the main structural features of cobinamides that lead to a differentiation between their α‐ and β‐faces. Only the ‘angular’ methyl group at C1 at the ring A/d‐junction may induce a notable deviation from the near‐*C*_2_‐symmetry of the corrin core. However, the rather small, computed deviation of the Co^II^ center from the plane of the corrin nitrogens in Cbi^+^ argues against a large effect of this methyl group on the corrin core. We believe that the remaining seven methyl groups do not play a significant role in modulating the Co_α_‐C and Co_β_−C BDE differently. We consider the influence exerted by the three acetamide and four propionamide side‐chains to be more important. The three acetamide side‐chains are all situated on the β‐face of the corrin ring. None of these engage in any significant interaction with the aryl group in the αArCbi^**+**^ structures, the aryl moiety being on the wrong side of the corrin for an interaction. In the βArCbi^**+**^ structures, one N−H bond from the acetamide side‐chain on ring B (see Scheme 1 for ring labeling) appears to interact strongly with aryl moiety's π‐system, the distance from the N−H hydrogen to the centroid of the aryl ring being computed to be only 2.40 Å . The other two acetamides in the βArCbi^**+**^ structures, on rings A and D, are hydrogen‐bonded with each other, and hence oriented well away from the aryl moiety. Analogously, but much more importantly, the four longer propionamide side‐chains, all situated on the α‐side of the corrin, engage in a much richer palette of interactions. In the βArCbi^**+**^ structures, and in the Cbi^+^ cation left after Co−C bond cleavage, the propionamide side chains engage in a network of hydrogen‐bonds, which remains largely invariant as one cleaves the aryl moiety on the other side of the corrin ring. For the αArCbi^**+**^ structures, however, the aryl moiety sits right in the middle of what would have been the hydrogen‐bond network. Examination of the computed structure of αEtPhCbi^**+**^, for example, finds disruption of the hydrogen bonds, and, furthermore, several novel, non‐covalent interactions of the propionamide side‐chains with the aryl moiety. Most prominent are close contacts, presumably non‐polar, between CH moieties on the propionamides on ring C and D, and the π‐system of the aryl moiety (2.90 and 2.41 Å, contacts 5 and 6 in Figure 5, left structure). Additionally, the computed structure finds close contacts of the ethyl group in the αEtPhCbi^**+**^ with the propionamide on ring D as well (2.69 Å). It appears legitimate to claim a high degree of congruence in the network of non‐covalent interactions for βArCbi^**+**^ and Cbi^+^, and a large incongruence for αArCbi^**+**^ and Cbi^+^. At the very least, the computational results for the corrins, *including all of the side‐chains*, underlines the importance of those side‐chains for a computed BDE, as the re‐shuffling of the non‐covalent interactions enters into the bond strength, by definition. Computations that truncate the side‐chains around the periphery of the corrin ring miss these effects altogether. A cartoon representing an admittedly oversimplified, but nevertheless instructive, model is given in Scheme 3. In the cartoon, we neglect the acetamide side‐chains on the β‐face and focus on the propionamide side‐chains on the α‐face.

The cartoon serves to emphasize the central results from the present experimental and computational findings. In the absence of solvent, the computed structures for conformers of the α‐diastereomers show evidence for stabilizing, non‐covalent interactions (depicted in blue in Scheme 3) between the side chains and the EtPh or Ph moieties, respectively. When the ion undergoes Co−C bond cleavage and removal of the hydrocarbon moiety, these interactions are replaced by *different* stabilizing, non‐covalent interactions among the side chains themselves (depicted in red in Scheme 3). Although one might expect that the *intrinsic* BDEs for the α‐ or β‐diastereomers should be similar, at least on electronic grounds (see above), it should be evident from Scheme 3, that the BDE of the β‐diastereomer could more closely represent that intrinsic bond strength for Co bound to an sp^2^‐hybridized carbon (aside from the NH–π interaction seen in Figure 5), because the red interactions in the β‐diastereomer (before cleavage) may approximately cancel out the red interactions in the corrin after cleavage, making the absolute magnitude of the red interactions immaterial with regard to that BDE. Considering further that both α‐ and β‐cleavages produce the same Co^II^‐corrin fragment, with the side chains tucked‐in to maximize hydrogen bonding with each other, the ground state energy differences for the α‐ and β‐ diastereomer should therefore correspond to the observed difference in the BDE between the two isomers. If “blue” interactions in the α‐diastereomer before cleavage are energetically better than the “red” interactions in the corrin moiety formed after the cleavage, then the BDE of the α‐diastereomer will be larger than the BDE for the β‐diastereomer, but if the “blue” interactions are smaller than the “red”, the order of BDEs may be reversed. In other words, unlike in the case of the β‐diastereomer, the absolute magnitudes of both the “blue” and the “red” interactions count for the BDE in the α‐diastereomer. Moreover, depending on which set of interactions is larger, one can, in principle, have an α‐BDE greater or lesser than the β‐BDE, or vice‐versa, the balance of the “blue” versus “red” in the cleavage of the α‐diastereomers becoming a sensitive measure of the treatment of the numerous non‐covalent interactions in a complicated system. We accordingly suggest that the reversal of the α‐ versus β‐BDEs in the experiment, as compared to the DFT predictions, is most probably due to an inadequate treatment of the large number of non‐covalent intramolecular interactions, certainly in the ground state of the α‐diastereomers, but perhaps also in the β‐diastereomers and homolysis product Co^II^‐corrin, although for the latter case, a good or bad treatment of the “red” interactions should make a far smaller difference for that BDE. The size and flexibility of the side‐chains in cobinamides with large, cleavable groups interacting with those side chains situated on the periphery of the corrin ring makes the computational modeling of the interactions extraordinarily challenging.

If one were to accept that the quantitative treatment of non‐covalent interactions is difficult, then the cancelation of the “red” interactions in dissociation of the β ‐isomers should mean that the latter BDE is potentially a better measure of the adequacy of the tested DFT methods for the Co−C bond. Disappointingly, comparison of Table 1 and Table 2 find that the experimental β‐BDEs differ from the computed values by about 16 kcal mol^−1^ for BP86 and 19 kcal mol^−1^ for BP86‐D3. It has been suggested earlier that a homolytic Co−C bond breaking could be an inherently multireference situation, which could make treatment by DFT problematic; CASSCF/CASPT2 calculations might provide for a better agreement between theory and experiment. Earlier attempts on CASSCF/CASPT2 calculations in methylcobalamin were performed by the Kozlowski group. With the largest affordable active space of (11,10) the calculations yielded 20.1 kcal mol^−1^ in the CASSCF case and 58.8 kcal mol^−1^ for CASPT2 for the Co‐Me bond, the numbers bracketing the experimental determination for methylcobinamide.^[11c]^ The CASSCF and CASPT2 numbers, relative to the single‐determinantal HF calculations, and relative to each other, indicate that both static and dynamic correlation are important for the Co−C bond. The known propensity of MP2 to overbind is also consistent with the CASPT2 value overshooting the experimental measurement. The Kozlowski group pointed out, however, that the discrepancies are most likely caused by the inadequate choice of active space, a larger active space becoming prohibitively expensive. This issue will require either substantially more computational resources, or new theory. It is important, in judging the plausibility of both the CASPT2 value and the higher DFT results, especially the BP86‐D3 BDEs, to consider what one would see in an experiment if the Co−C${}_{\mathrm{sp}{}^{2}}$ were really nearly 70 kcal mol^−1^ (Table 2). In large, flexible molecules, there are other bonds with a BDE in the 60–80 kcal mol^−1^ range, and competitive cleavages would appear in gas‐phase CID experiments if enough energy is imparted by collision to cleave a Co−C${}_{\mathrm{sp}{}^{2}}$ bond with BDE close to 70 kcal mol^−1^. Indeed, when we initially started to investigate cobalamin structures in the gas phase, we needed to prune back such side chains to get a clean CID for threshold CID measurements, which led to the synthesis of cobinamides investigated here. We note in passing that, in those structures there is still one remaining, longer amide side chain that could be cleaved, if the collision energy exceeded ca. 50 kcal mol^−1^ (as estimated from both DFT calculations and l‐CID experiments on the structures that involve such a side chain), but which remains intact under the experimental conditions. This observation alone does not say that the Co−C${}_{\mathrm{sp}{}^{2}}$ bond has an “inherent” BDE around 40 kcal mol^−1^, but it does argue strongly against one as strong as 70 kcal mol^−1^.

Earlier gas‐phase experiments reported by Kobylianskii et al. pointed to a problem, for which the present results suggest a resolution. We reported that some of the popular functionals could reproduce the experimental BDEs for either AdoCbi^+^ and MeCbi^+^, but none could reproduce both.^[17]^ The most recent, and extensive, attempt to calculate BDEs of cobalamins with DFT matched our methylcobinamide values well enough, but they failed to reproduce the experimental numbers found by the Chen group for adenosylcobinamide, for which we remark that the cleaved adenosyl group is large and conformationally flexible.^[11d]^ It should be emphasized, though, that the same study calculated a difference of the BDEs between methylcobalamin and methylcobinamide in water (6.8 kcal mol^−1^ weaker bond in methylcobalamin), which is, in fact, in the reverse order from that found experimentally by Kräutler in a direct equilibration experiment in aqueous solution.^[15]^ Returning to the gas‐phase experiment, the discrepancy to computed BDEs could be interpreted as casting doubt on the ability of modern DFT methods to describe large systems accurately, in general. It also suggested that current implicit solvent models may introduce new levels of uncertainty.^[51]^ Given a further discrepancy between experimentally determined bond energies in the present study, and the corresponding computed values, one should always consider, as a matter of principle, that one, the other, or perhaps even both, could be wrong. In this context, we have further investigated the possibility that the discrepancy could arise due to a failure in either the threshold CID experiment, or the deconvolution of the experimental data by the l‐CID program. To this end, we designed control experiments with several model systems to test the scaling of the experimental methods with the size of the molecule, and also to isolate the contribution of London dispersion,^[52]^ the latter non‐covalent interaction being the object of the D3 correction applied to DFT calculations. The control experiments revealed that the l‐CID deconvolution of the T‐CID experiment operates correctly for both small and large ions within the tested range and agrees with the calculated BDEs, with the fairly trivial proviso that the structures of the initial ion, as well as its dissociation products, have to be correct. Turning to potential issues with the computational results, the present results suggest a resolution of the previously observed discrepancy: good agreement with MeCbi^+^ and poor agreement with AdoCbi^+^. An insufficiently accurate consideration of the interactions among the side‐chains themselves, and between the side‐chains and the cleavable group on Co, most certainly impacts the result for the large adenosyl moiety, with its conformational flexibility, its hydrogen‐bonding functionalities, and its extended π‐system, all presenting possibilities for non‐covalent interactions. Accordingly, one might expect an adequate agreement of the gas‐phase experiment on MeCbi^+^ with a computed BDE for MeCbi^+^ with or without sufficient consideration of the side‐chains. A correspondingly poorer agreement upon neglect of the side‐chains in the case of AdoCbi^+^ would, hence, make sense. We should point out that, independent of whether or not l‐CID delivers a quantitatively accurate extraction of *E*_0_ from the T‐CID curves, the plain observation that αArCbi^+^ and βArCbi^+^ have significantly different BDEs in the gas phase, Figure 4, shows that the side‐chains around the periphery of the corrin must be carefully treated in a minimally adequate computational model. Lastly, the computational study by Kepp for AdoCbl reports similar issues with the side‐chains.^[49b]^

One may consider hypothetical consequences of a conclusion that the intrinsic Co−C bond dissociation energy may be significantly modified by the net change in non‐covalent interactions involving the sidechains spaced around the periphery of the corrin ring. As had been cited in the Introduction, there have been a range of determinations of the Co−C BDEs in solution, mostly by kinetic measurements, as well as our previous, and present, determinations in the gas phase.^[18]^ Going from the gas phase into aqueous solution, the BDEs are reduced significantly, if one considers the known cases of MeCbi^+^ and AdoCbi^+^, for which only the β‐diastereomers have been investigated (as discussed above). The data obtained in the present work for the α‐ vs. β‐phenylcobinamide diastereomers indicate an apparent reversal of the relative order of the BDEs and, hence, of the relative ArCbi^+^‐isomer stability. Considering the important role of the intramolecular non‐covalent interactions in the gas phase, one should perhaps not be surprised that large changes in BDE occur upon transfer into solution, as the intramolecular interactions may be screened or compensated, in whole or in part, by the medium.^[51a]^ More importantly, in solution, the intramolecular non‐covalent interactions among the sidechains compete with comparable intermolecular interactions of these same sidechains with polar (aqueous) solvent molecules, which leads, for example, to a less tucked‐in structure for the strongly hydrated, related corrin species in aqueous solution and in their crystals.[^9d^, ^20^] These changes, according to the hypothesis advanced above, will certainly impact the overall thermochemical balance for Co−C bond cleavage. Considering further that, in the enzyme, the cofactor finds itself bound in an environment with an ordered array of groups that interact with the sidechains on the corrin non‐covalently, the array of such non‐covalent interactions will not only provide a tight binding interface, but also contribute, potentially, to the yet further reduced BDE of adenosylcobalamin (AdoCbl) in the enzyme.[^19^, ^49b^] Thus, the non‐covalent interactions involving the bound cofactor must not be neglected as a factor in possibly modulating the dissociation energy of the Co−C bond of the B_12_‐cofactor AdoCbl. The network of interactions that we observe to play a role in the gas phase thermochemistry of the non‐natural aryl‐corrinoids accordingly suggests means for the modification of the BDEs for the natural corrinoid cofactors by an enzyme environment, or by solvation.

## Conclusions

Our study indicates a counterintuitively smaller homolytic Co−C BDE (in the gas phase) for the Co−C${}_{\mathrm{sp}{}^{2}}$ bond in aryl‐cobinamides than for the Co−C${}_{\mathrm{sp}{}^{3}}$ bond in the corresponding methylcobinamide. Accordingly, in contrast to what had been proposed originally,^[20a]^ the strength of the Co−C${}_{\mathrm{sp}{}^{2}}$ bond of the antivitamin B_12_ EtPhCbl is not a major factor responsible for its essential resistance towards enzymatic tailoring. As discussed above, the inactivity of EtPhCbl is due, rather, to the specific geometric and mechanistic boundary conditions imposed on this enzyme catalyzed nucleophilic substitution reaction.[^43^, ^44^] Our study also presents the first experimental gas phase data on isomeric Co^III^‐corrins with an organometallic ligand at either one of the two diastereotopic axial coordination sites. It has revealed an effectively stronger Co_α_−C bond to an aryl group, than a corresponding Co_β_−C bond and, consequently, the higher stability of the α‐isomer compared to the β‐isomer. These gas‐phase results are surprising, considering the higher stability of the Co_β_‐isomer of the methylcobinamides and other methylcobyrinates in aqueous solution.[^15^, ^40^] However, we identified a series of non‐polar interactions with the axial cobalt‐bound aryl group, as well as H‐bonding contributions in the periphery of the cobinamide moiety, which could potentially be responsible for the apparent ‘stability reversal’ in the gas phase. When compared with experimental or calculated data referring to chemistry in (aqueous) solution, the additional specific effects of the polar solvent come into play, for example, as a possible ligand at the corrin‐bound Co^III^‐ or Co^II^‐centers, or from the effective interaction of water with the polar groups at the periphery of the natural cobinamide core. Clearly, similar but more specific effects by the full binding interface with the protein environment, which are an often still incompletely resolved feature in an evolved enzyme, also need to be taken into account when interpreting the means of the protein part of the B_12_‐dependent enzymes to activate the bound B_12_‐cofactors and to steer their biologically important reactivity.

## Conflict of interest

The authors declare no conflict of interest.

## Supporting information

As a service to our authors and readers, this journal provides supporting information supplied by the authors. Such materials are peer reviewed and may be re‐organized for online delivery, but are not copy‐edited or typeset. Technical support issues arising from supporting information (other than missing files) should be addressed to the authors.

Supplementary
