# Supplementary material for: Surprising Homolytic Gas Phase Co−C Bond Dissociation Energies of Organometallic Aryl‐Cobinamides Reveal Notable Non‐Bonded Intramolecular Interactions
Source: Chemistry. 2021 Mar 25;27(25):7252–64. doi: 10.1002/chem.202004589 (PMC8251903; doi:10.1002/chem.202004589)
Supplement: Supplementary file 1 — Supplementary [file CHEM-27-7252-s001.pdf]

Chemistry–A European Journal

Supporting Information

Surprising Homolytic Gas Phase Co−C Bond Dissociation Energies of Organometallic Aryl-Cobinamides Reveal Notable Non-Bonded Intramolecular Interactions

Alexandra Tsybizova+,[a] Christopher Brenig+,[b] Christoph Kieninger,[b] Bernhard Kräutler,*[b] and Peter Chen*[a]

Electronic Supporting Information for

Surprising Homolytic Gas Phase (Co-C)-Bond Dissociation Energies of Organometallic Aryl-Cobinamides Reveal Notable Non-Bonded Intramolecular Interactions

Alexandra Tsybizova,[†,¶] Christopher Brenig,[‡,¶] Christoph Kieninger,[‡] Bernhard Kräutler,[*,‡] and Peter Chen[*,†]

†*Laboratorium für Organische Chemie, Department of Chemistry and Applied Biosciences, ETH Zürich, Zürich, Switzerland*

‡*Institute of Organic Chemistry & Center of Molecular Biosciences, University of Innsbruck, Innsbruck, Austria*

¶*Contributed equally to this work*

E-mail: Bernhard.Kraeutler@uibk.ac.at; peter.chen@org.chem.ethz.ch

Experimental Part

Synthesis and Spectroscopy

Materials and Methods

Dicyano-cobinamide was prepared according to ref,[1] purified by column chromatography and by preparative reversed phase MPLC ($40-50\%$ yield). Water was purified using an *Epure* water purification system from *Barnstead Co*, diphenyliodonium chloride was obtained from Sigma-Aldrich, di(4-ethylphenyl)iodonium tetrafluoroborate was synthesized according to ref.[2] and re-crystallized from aqueous solution (50% yield). Sodium borohydride, sodium tetra fluoroborate (reagent grade, *Sigma-Aldrich*) and methanol (gradient grade, *VWR Chemicals*) were used as received. If not otherwise stated, all reactions were carried out under argon atmosphere. UV-Vis (λ_{max} in nm (log ϵ), in H_2O): *Agilent Cary* 60; ESI-MS (m/z (%), pos. ions, in H_2O/MeOH): *Finnigan LCQ classic*, ESI spray voltage: 4.2 kV. ^{1}H-NMR (δ (ppm) relative to $\delta_{HDO} = 4.75$ ppm, J in Hz, in D_2O, 298 K): *Varian Unity Inova* 500, equipped with 5 mm triple-resonance probe with z-gradients. ^{13}C-NMR spectra (δ in ppm) data from ^{1}H,^{13}C-heteronuclear correlations. Chemical shift and signal assignment were based on ^{1}H, (^{1}H,^{1}H)-COSY, (^{1}H,^{1}H)-ROESY, (^{1}H,^{13}C)-HSQC and (^{1}H,^{13}C)-HMBC spectra. ^{11}B-NMR (128 MHz) and ^{19}F-NMR (376 MHz): 400 MHz Bruker Avance 4 Neo spectrometer, at 298 K. MPLC system: *Büchi Pump Manager C 615* and *Pump Module C 605*, flow rate: 10 ml/min, column: 100 g *LiChroprep (Merck)* RP-18 (24-40 μm; manually packed), detection wavelength: 280 nm. HPLC system: *Dionex UltiMate 3000*, *Phenomenex HyperClone* ODS (C18) column, mobile phase A: 10 mM phosphate buffer pH 7; B: MeOH, 5-95% B in 40 min. The syntheses of the aryl-cobinamides and their purification were carried out with protection from light.

Procedure for the synthesis of $Co_{\alpha/\beta}$-aryl-cobinamide tetrafluoroborates: In a 25 ml flask, 25 mg of dicyano-cobinamide (CN_2Cbi, 24.0 μmol) were dissolved in H_2O (4mL) and 25 l acetic acid were added. The mixture was stirred at 25 °C and HCN formed was removed

by application of a slight vacuum; a reddish color developed, indicating the formation of aquo,cyano-cobinamide (H_2O,CNCbi$^+$). The red solution was loaded on a conditioned and equilibrated *SepPack*Ⓡ cartridge and acetic acid was washed out with H_2O (20 ml). The cobinamide fraction was eluted with methanol and after removing the solvent under reduced pressure, re-dissolved in H_2O (4 mL). The solution was transferred to a *Schlenk* flask and degassed, using freeze pump thaw cycling. Afterwards, sodium borohydride (9.1 mg, 240 μmol) were added under argon counter flow and the mixture was stirred at 25 °C for 60 min. The resulting brown to violet solution was protected from light and the diaryliodonium salt ($(EtPh)_2IBF_4$ or $(Ph)_2ICl$ 48.0 μmol, 2.0 equiv.) was added. After 45 min of stirring, 9.1 mg (240 μmol) of additional sodium borohydride was added, followed by stirring for 30 min and further addition of the diaryliodonium salt (48.0 μmol. After 125 min of total reaction time, reaction control via HPLC showed full consumption of the starting material. The reaction mixture was diluted by addition of 10 ml of H_2O and was loaded on a conditioned and equilibrated *SepPack*Ⓡ cartridge. After desalting with H_2O (20 ml), the products were eluted with methanol and the solvent was evaporated under reduced pressure. The residues were purified using preparative semi-automated reversed phase MPLC. Fractions containing corrinoid material were analyzed using HPLC. Pure fractions of Co_α-ArCbis and Co_β-ArCbis, respectively, eluted in this order and were pooled. Solvents were removed on a rotary evaporator and the residues were desalted using *SepPack*Ⓡ catriges. The absorbed orange products were washed with 10 ml of a 0.1 M sodium tetrafluoroborate solution and eluted with methanol/aq. 0.1 M $NaBF_4$ solution (9:1) as BF_4-salts. Co_α-ArCbis were precipitated from methanol with ethyl acetate and dried, affording 16.2 mg of Co_α-phenyl-cobinamide tetrafluoroborate (Co_α-PhCbi[BF_4] = αPhCbi, 58%) or 17.8 mg of Co_α-(4-ethylphenyl)-cobinamide tetrafluoroborate (Co_α-EtPhCbi[BF4] = αEtPhCbi, 62%), respectively, as orange powdery solids. The purified Co_β-ArCbis were precipitated from methanol with MTBE and dried, affording 6.8 mg of Co_β-phenyl-cobinamide tetrafluoroborate (Co_β-PhCbi[BF_4] = βPhCbi, 24%) or 5.9 mg of Co_β-(4-ethylphenyl)-cobinamide tetrafluoroborate (Co_β-EtPhCbi[BF4] =

βEtPhCbi, 21%), respectively, as yellow powdery solids.

Co$_\beta$-phenyl-cobinamide tetrafluoroborate (βPhCbi). **UV-VIS** (c=0.043 mM): 264 (4.39), 296 (4.36), 328 (4.29), 392 (3.95), 453 (4.04), 484 (3.95). **ESI-MS**: 1069.6 (11), 1068.5 (51), 1067.5 (72), 1066.5 (100, M^+). ^{1}**H-NMR** (c=8.5 mM): 0.93 (s, 3H, H_3C12B), 1.10 – 1.15 (m, 6H, H_3C1A, H_3C177), 1.24 (s, 3H, H_3C17B), 1.45 (s, 3H, H_3C2A), 1.57 (s, 3H, H_3C12A), 1.76 (d, 1H, J = 13.9 Hz, H_aC71), 1.83 (s, 3H, H_3C7A), 1.84 – 1.98 (m, 5H, H_aC21, C32, C81, C171, C131), 1.99 – 2.11 (m, 4H, H_aC31, C132, H_bC21, C171), 2.18 – 2.35 (m, 5H, H_bC81, C31, C131, C132, H_aC82), 2.40 – 2.47 (m, 1H, H_bC82), 2.49 – 2.67 (m, 12H, H_bC71, C32, H_2C181, C172) superimposed by 2.56 (s, 3H, HC51) and 2.57 (s, 3H, HC151), 2.90 – 2.98 (m, 1H, HC18), 3.16 (dd, 1H, J = 13.9, 4.4 Hz, H_aC175), 3.24 (dd, 1H, J = 13.9, 4.4 Hz, H_bC175), 3.63 (d, 1H, J = 2.2 Hz, HC13), 3.69 (dd, 1H, J = 8.1, 4.4 Hz, HC8), 3.76 (d, 1H, J = 10.3 Hz, HC19), 3.86 - 3,91 (m, 1H, HC176), 4.25 (d, 1H, J = 8.8 Hz, HC3), 5.10 (d, 2H, J = 8.1 Hz, HC2L, C6L), 6.62 (t, 2H, J = 7.7 Hz, HC3L,C5L), 6.66 (s, 1H, HC10), 6.75 (t, 1H, J = 7.7 Hz, HC4L); see Table S1 for ^{13}C NMR assignments; ^{11}B-NMR (c = 1.84 mM): δ = -1.45 (quint, J(F) = 1.2 Hz) ^{19}F-NMR (c = 1.84 mM): δ = -150.06 ($^{10}BF_4$, bs, half width = 4 Hz), δ = -150.11 ($^{11}BF_4$, bs, half width = 6 Hz)

Co$_\alpha$-phenyl-cobinamide tetrafluoroborate (αPhCbi). **UV-VIS** (c=0.043 mM): 264 (4.34), 300 (4.29), 333 (4.33), 465 (4.05), 490 (4.08). **ESI-MS**: m/z (%) = 1069.5 (7), 1068.4 (22), 1067.4 (66), 1066.5 (100, M^+). ^{1}**H-NMR** (c=8.5 mM): 0.78 (s, 3H, H_3C1A), 1.13 (d, 3H, J = 6,6 Hz, H_3C177), 1.26 – 1.32 (m, 6H, H_3C12B, -17B), 1.42 (s, 3H, H_3C12A), 1.45 (s, 3H, H_3C2A), 1.54 (ddd, 1H, J = 15.6, 10.1, 5.1 Hz, H_aC81), 1.62 – 1.70 (m, 2H, H_2C131), 1.70 – 1.75 (m, 1H, H_bC81), 1.78 (s, 3H, H_3C7A), 1.82 – 1.90 (m, 1H, H_aC171), 2.00 – 2.17 (m, 3H, HaC31, C172), 2.18 – 2.27 (m, 3H, H_2C21, H_bC31), 2.39 – 2.62 (m, 14H, H_2C32, C82, C132, H_bC171, C172) superimposed by 2.46 (s, 3H, H_3C51) and 2.55 (s, 3H, H_3C151), 2.63 – 2.70 (m, 4H, H_2C71, C181), 2.79 (dd, 1H, J = 11.0, 6.6 Hz, HC18), 3.11 – 3.19 (m, 1H, H_aC175), 3.22 – 3.28 (m, 1H, H_bC175), 3.39 – 3.47 (m, 2H, HC8, C13), 3.87 – 3.93 (m, 2H, HC3, C176), 3.96 (d, 1H, J = 11.0 Hz, HC19), 5.36 (d, 2H, J = 8.1 Hz, HC2L, C6L),

6.24 (s, 1H, HC10), 6.55 (t, 3H, J = 7.7 Hz, HC3L, C5L), 6.68 (t, 1H, J = 7.7 Hz, HC4L); see Table S2 for ^{13}C NMR assignments; ^{11}B-NMR (c = 1.84 mM): δ = -1.45 (quint, J(F) = 1.2 Hz); ^{19}F-NMR (c = 1.84 mM): δ = -150.06 ($^{10}BF_4$, bs, half width = 4 Hz), δ = -150.11 ($^{11}BF_4$, bs, half width = 6 Hz).

Co$_\beta$-(4-ethylphenyl)-cobinamide tetrafluoroborate (βEtPhCbi). **UV-VIS** (c=0.042 mM): 264 (4.37), 299 (4.34), 327 (4.25), 391 (3.91), 454 (4.00), 488 (3.88). **ESI-MS** m/z (%) = 1097.3 (8), 1096.5 (25), 1095.5 (77), 1094.4 (100, M^+). ^{1}**H-NMR** (c=8.3 mM): 0.92 (s, 3H, H_3C12B), 0.97 (t, 3H, J = 7.3 Hz, H_3C8L), 1.12 (br. s, 3H, H_3C1A), 1.15 (d, 3H, J = 6.6 Hz, H_3C177), 1.26 (s, 3H, H_3C17B), 1.47 (s, 3H, H_3C2A), 1.58 (s, 3H, H_3C12A), 1.73 (d, 1H, J = 13.9 Hz, H_aC71), 1.81 - 2.20 (m, 12H, H_2C21, C31, C171, H_aC32, C81, C132) superimposed by 1.85 (s, 3H, H_3C7A), 2.21 - 2.37 (m, 7H, H_2C7L, C131 H_bC81, C132, H_aC82), 2.40 - 2.49 (m, 1H, H_bC82), 2.50 - 2.70 (m, 12H, H_2C172, C181, H_bC32, C71) superimposed by 2.57 (s, 3H, H_3C51) and 2.59 (s, 3H, H_3C151), 2.91 - 3.01 (m, 1H, HC18), 3.18 (dd, 1H, J = 13.9, 4.4 Hz, H_aC175), 3.26 (dd, 1H, J = 13.9, 4.4 Hz, H_bC175), 3.65 (br. s, 2H, HC13), 3.69 - 3.76 (m, 2H, HC8), 3.82 (d, 1H, J = 10.3 Hz, HC19), 3.86 - 3.95 (m, 1H, HC176), 4.26 (d, 1H, J = 8.8 Hz, HC3), 5.00 (d, 2H, J = 8.1 Hz, HC2L, C6L), 6.53 (d, 2H, J = 8.1 Hz, HC3L, C5L), 6.69 (s, 1H, HC10).

Co$_\alpha$-(4-ethylphenyl)-cobinamide tetrafluoroborate (αEtPhCbi). **UV/Vis** (c=0.042 mM): 264 (4.31), 296 (4.23), 328 (4.26), 392 (3.74), 456 (3.97), 485 (4.03). **ESI-MS**: m/z (%) = 1097.5 (5), 1096.4 (23), 1095.5 (97), 1094.5 (100, M^+).^{1}**H-NMR** (c=8.3 mM): 0.83 (s, 3H, H_3C1A), 0.93 (t, 3H, J = 7.7 Hz, H_3C8L), 1.15 (d, 3H, J = 6.6 Hz, H_3C177), 1.30 (s, 6H, H_3C12B, C17B), 1.43 (s, 3H, H_3C12A), 1.47 (s, 3H, H_3C2A), 1.58 - 1.67 (m, 1H, H_aC131), 1.68 - 1.81 (m, 5H, H_aC81, HC131) superimposed by 1.78 (s, 3H, H_3C7A), 1.83 - 1.93 (m, 1H, H_aC171), 1.99 - 2.25 (m, 6H, H_2C21, C31, H_bC81, H_aC172), 2.30 (q, 2H, J = 7.3 Hz, H_2C7L), 2.37 - 2.63 (m, 14H, H_2C32, C82, C132, H_bC171, C172) superimposed by 2.47 (s, 3H, H_3C51) and 2.56 (s, 3H, H_3C151), 2.64 - 2.72 (m, 4H, H_2C181, C71), 2.78 - 2.85 (m, 1H, HC18), 3.16 (dd, 1H, J = 13.6, 7.0 Hz, H_aC175), 3.26 (dd, 1H, J = 13.9, 5.1 Hz, H_bC175), 3.40 - 3.49

(m, 2H, HC8, C13), 3.86 – 3.94 (m, 2H, HC3, C176), 3.96 (d, 1H, $J = 11.0$ Hz, HC19), 5.24 (d, 2H, J = 7.3 Hz, HC2L, C6L), 6.27 (s, 1H, HC10), 6.44 (d, 2H, $J = 7.3$ Hz, HC3L, C5L).

Collection of spectra and spectral data

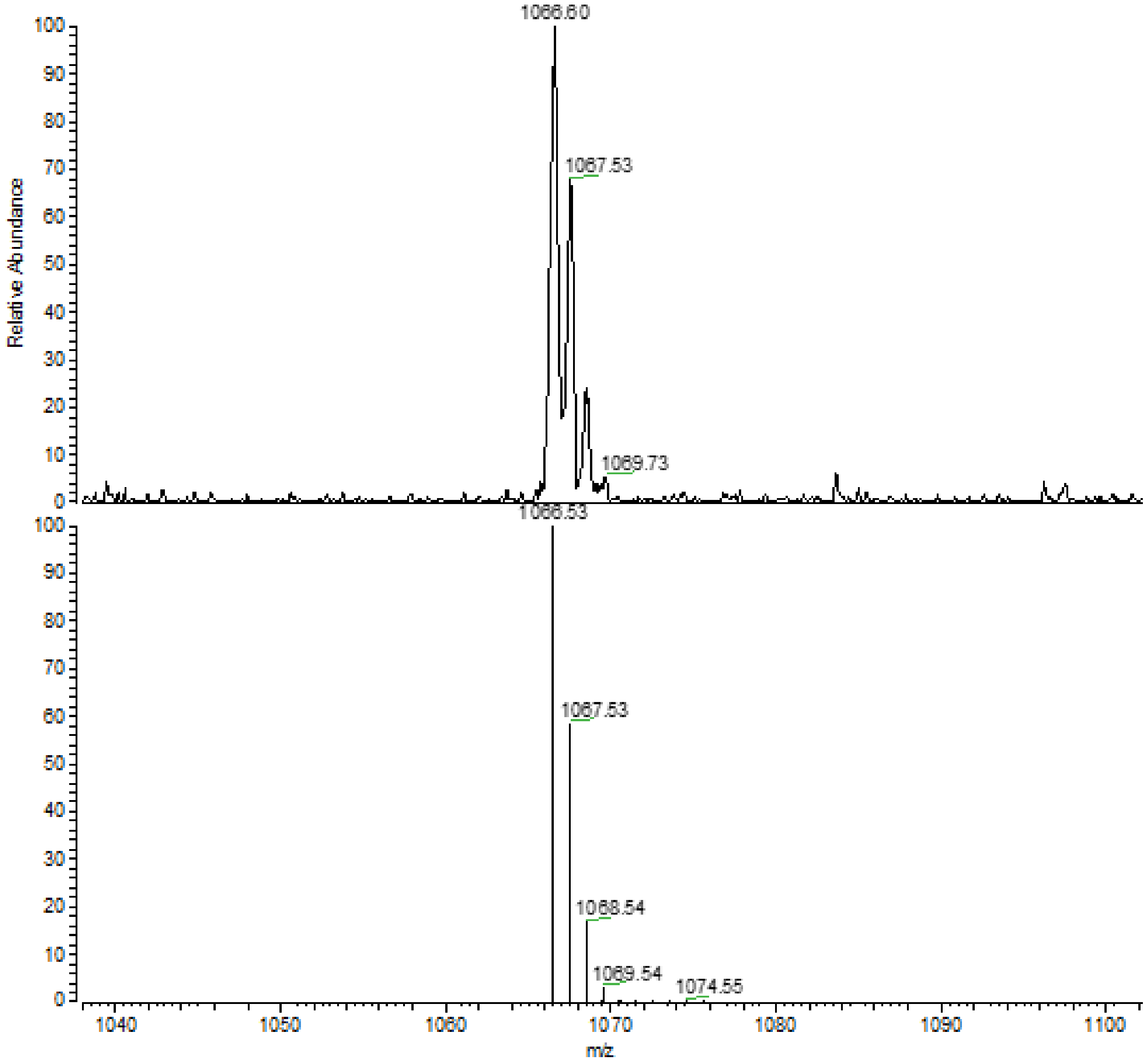

Figure S1: ESI-MS spectrum (pos. mode, MeOH/H_2O) of βPhCbi$^+$(top) and the simulated isotope pattern for $C_{54}H_{77}CoN_{11}O_8$ (bottom).

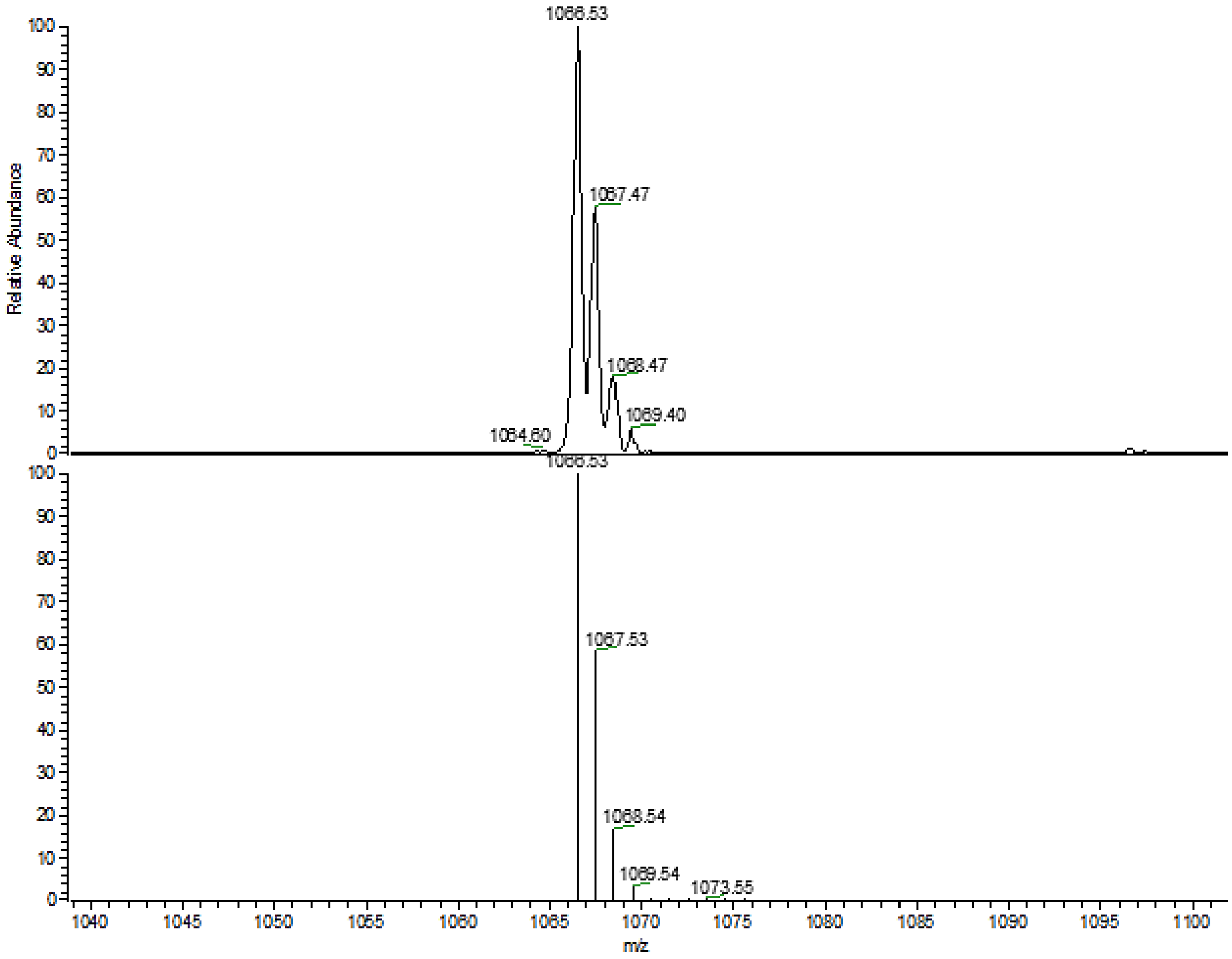

Figure S2: ESI-MS spectrum (pos. mode, MeOH/H_2O) of αPhCbi$^+$(top) and the simulated isotope pattern for $C_{54}H_{77}CoN_{11}O_8$ (bottom).

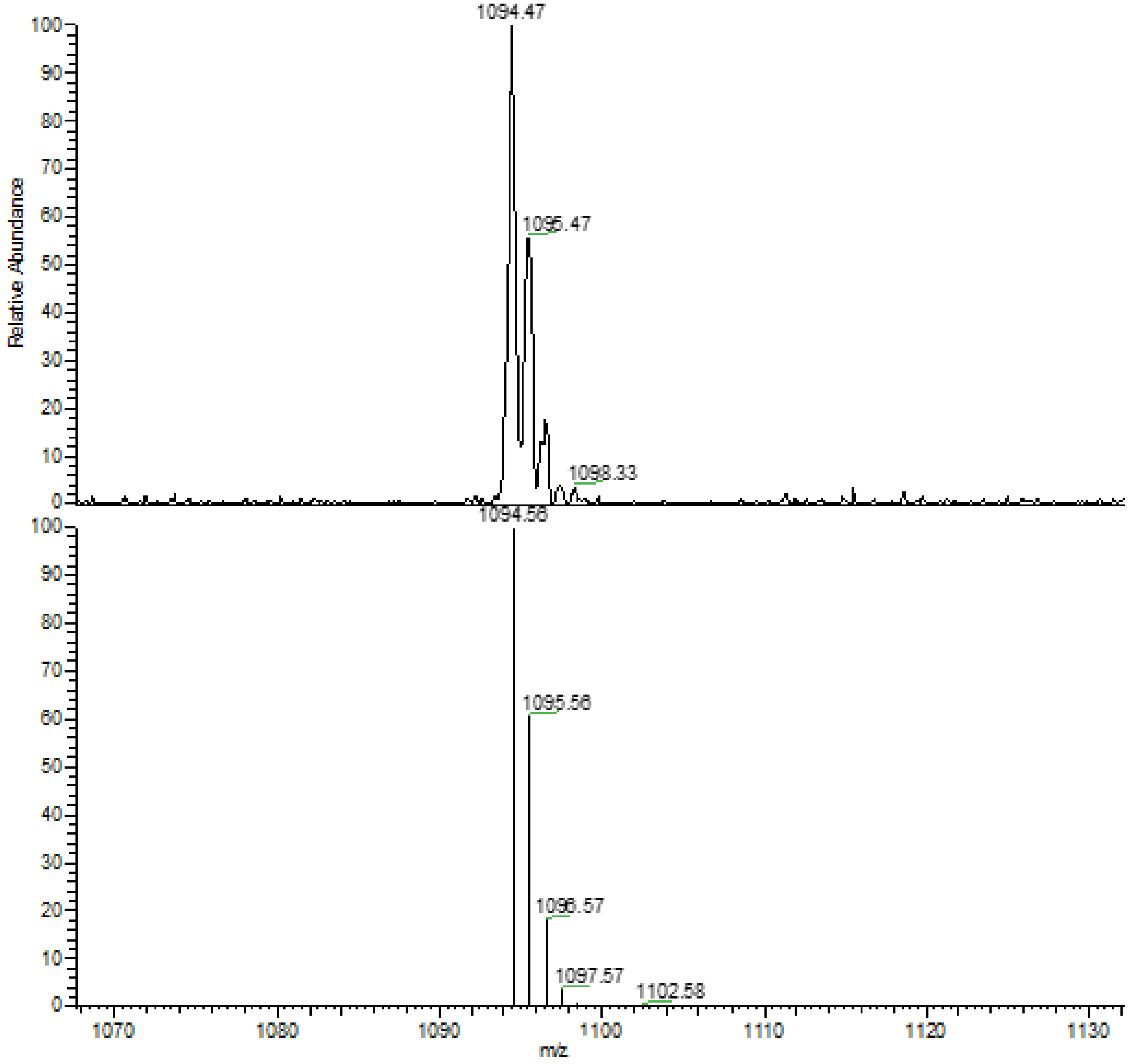

Figure S3: ESI-MS spectrum (pos. mode, MeOH/H_2O) of βEtPhCbi$^+$(top) and the simulated isotope pattern for $C_{56}H_{81}CoN_{11}O_8$ (bottom).

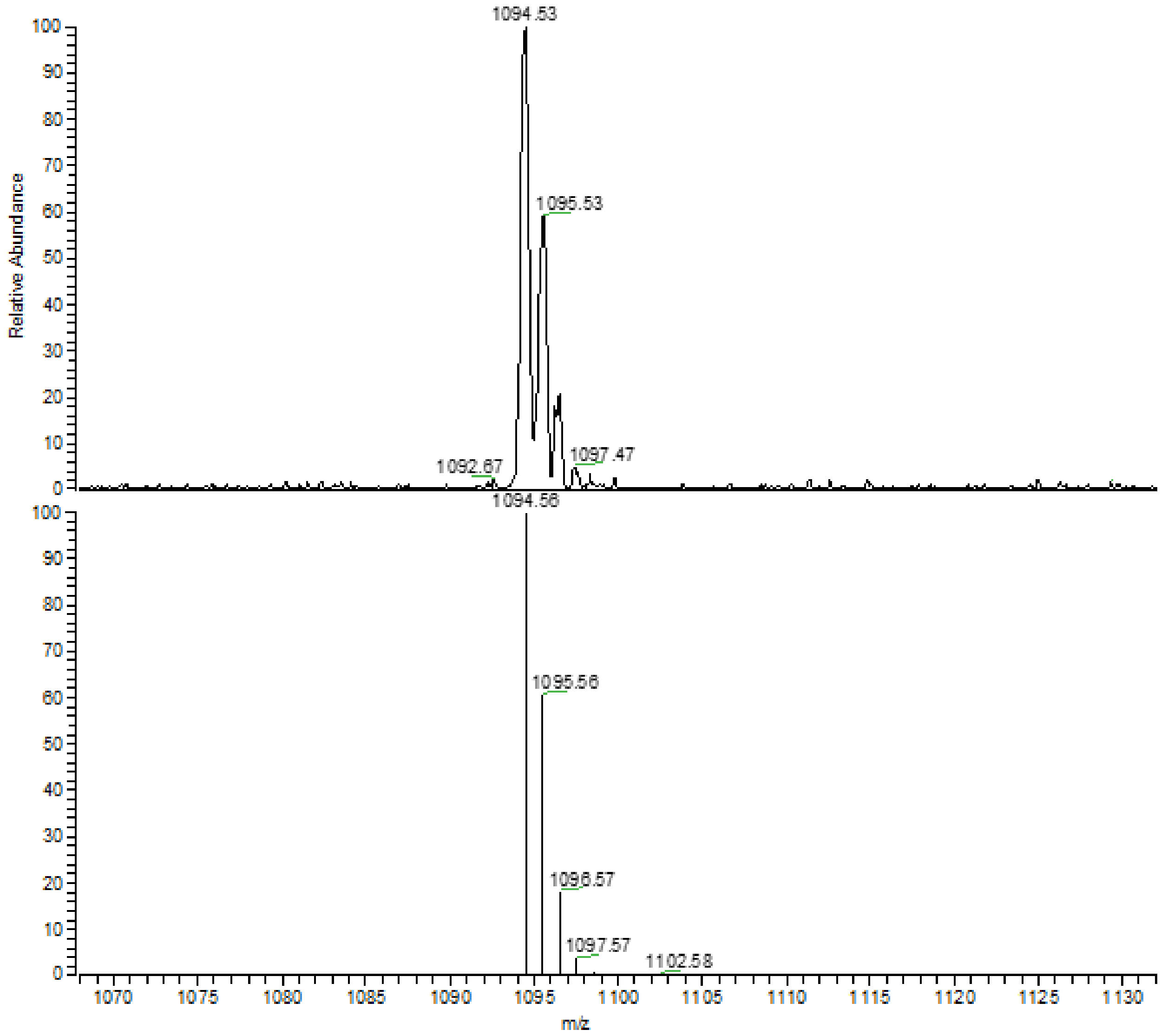

Figure S4: ESI-MS spectrum (pos. mode, MeOH/H_2O) of αEtPhCbi$^+$(top) and the simulated isotope pattern for $C_{56}H_{81}CoN_{11}O_8$ (bottom).

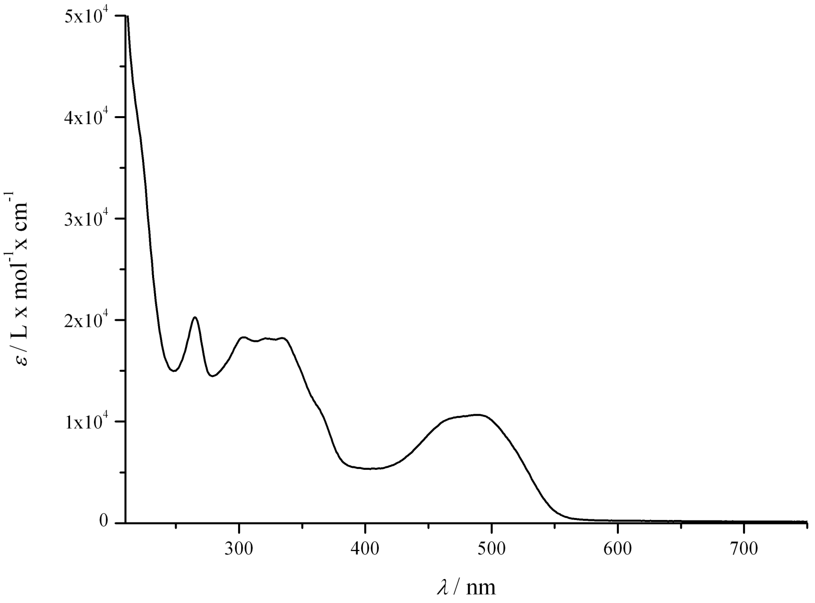

Figure S5: UV/Vis spectrum of αEtPhCbi ($4.3 \cdot 10^{-5}$ mol L^{-1}) in H_2O.

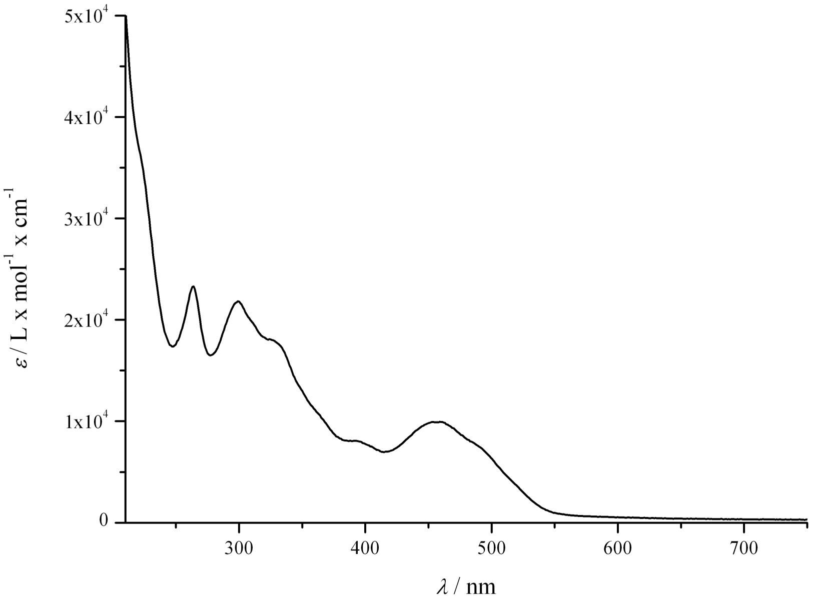

Figure S6: UV/Vis spectrum of βEtPhCbi ($4.2 \cdot 10^{-5}$ mol L^{-1}) in H_2O.

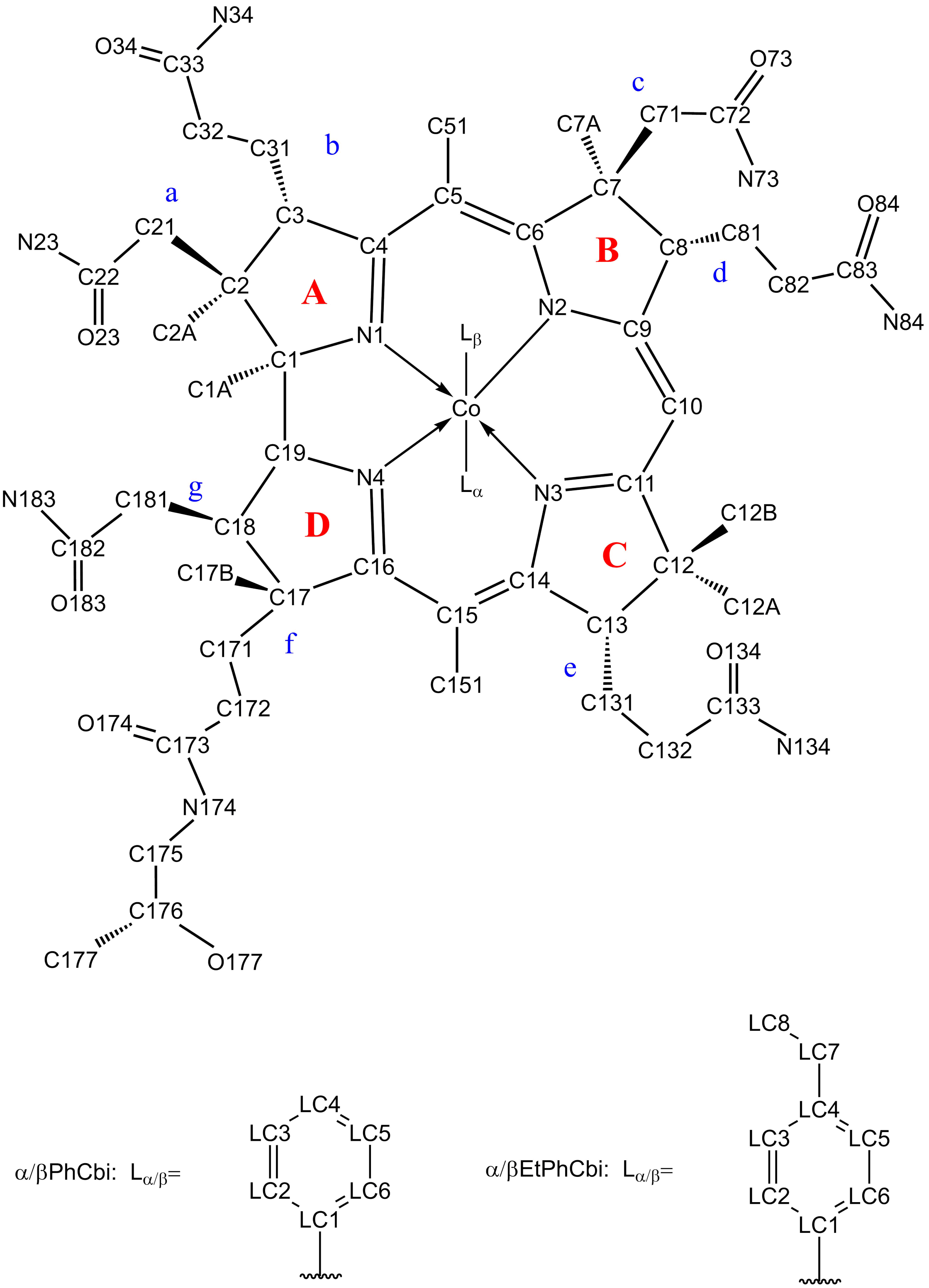

Figure S7: Atom numbering of arylcobinamides, including labels for 5-membered rings and amide side chains.

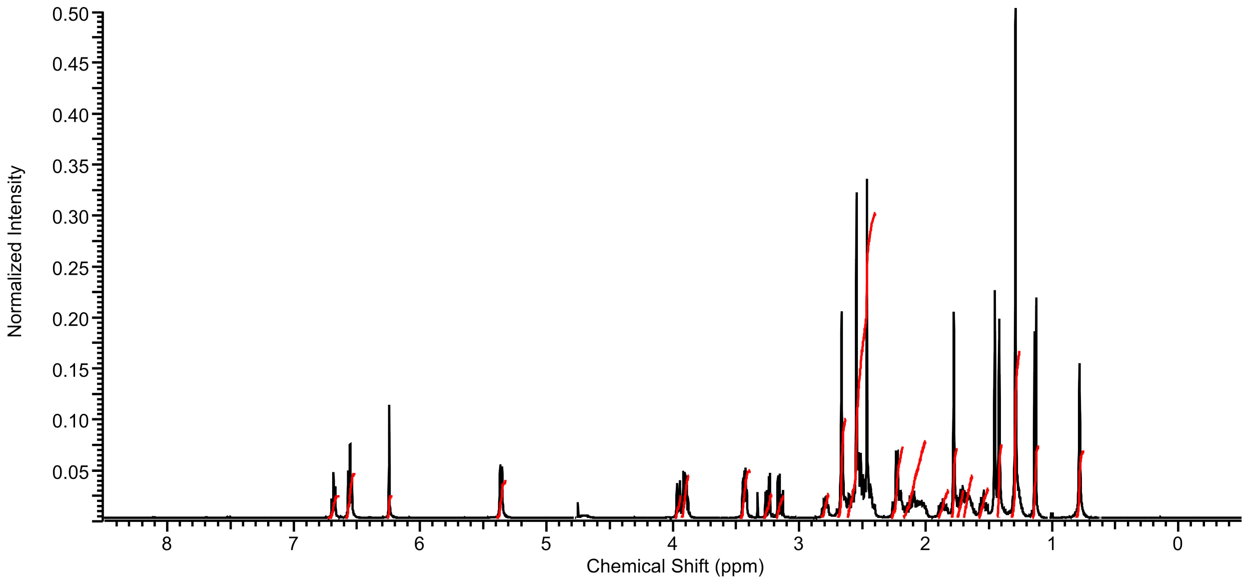

Figure S8: 500 MHz ^{1}H-NMR spectrum of αPhCbi ($4.3 \cdot 10^{-3}$ mol L^{-1}) in D_2O at 298K.

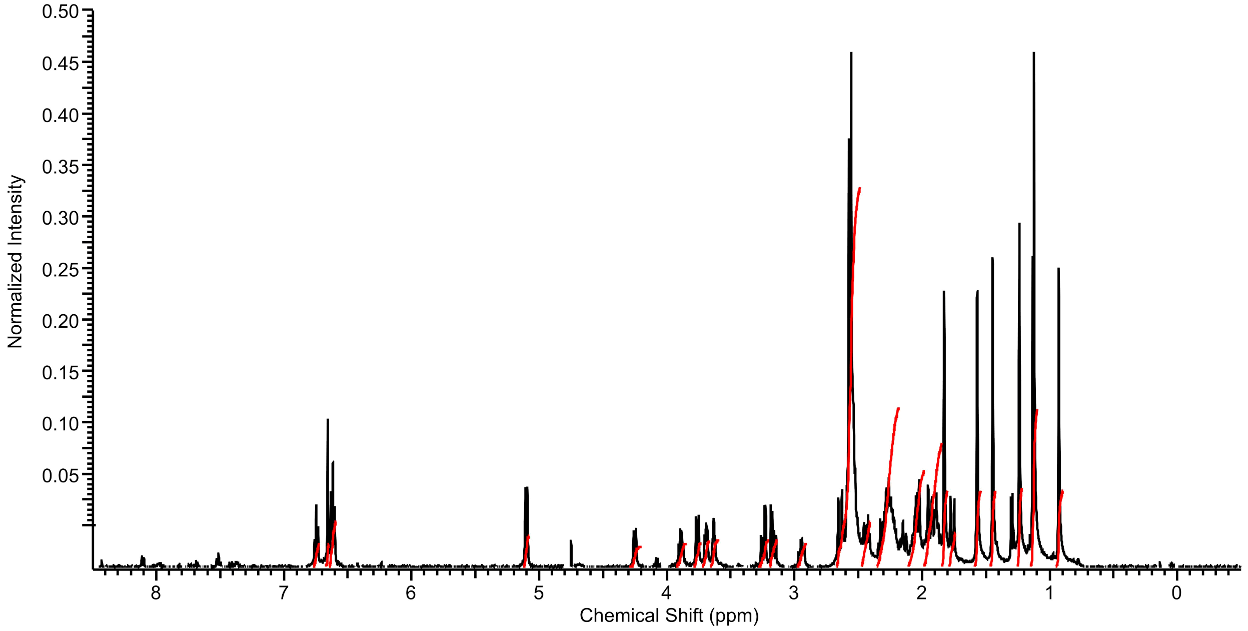

Figure S9: 500 MHz ^{1}H-NMR spectrum of βPhCbi ($4.2 \cdot 10^{-3}$ mol L^{-1}) in D_2O at 298K.

Table S1: Assignment of all non-exchangeable ^{1}H and all ^{13}C chemical shifts in αPhCbi

**assignment**	δ^{13}**C (ppm)**	δ^{1}**H (ppm)**	**Nprot**
C1A	19.5	0.78	3
C12B	27.5	1.29	3
C81	28.1	1.54/ 1.72	2
C17B	13.5	1.29	3
C82	31.3	2.51	2
C177	15.6	1.13	3
C2A	13.3	1.45	3
C12A	15.4	1.42	3
C71	41.5	2.66	2
C171	29.5	1.86/ 2.61	2
C7A	17.0	1.78	3
C21	39.2	2.22	2
C31	21.5	2.06/ 2.22	2
C131	23.5	1.67	2
C172	28.1	2.12/ 2.43	2
C181	28.5	2.66	2
C32	30.1	2.46/ 2.54	2
C132	31.3	2.51	2
C151	12.3	2.55	3
C18	35.6	2.79	1
C51	12.6	2.46	3
C175	42.3	3.15/ 3.25	2
C13	49.9	3.43	1
C19	72.3	3.95	1
C8	52.2	3.43	1
C3	52.7	3.9	1
C176	62.4	3.9	2

Table S1: Assignment of all non-exchangeable ^{1}H and all ^{13}C chemical shifts in αPhCbi

assignment	δ^{13}C (ppm)	δ^{1}H (ppm)	Nprot
C10	91.1	6.24	1
C2	42.8		0
C12	43.5		0
C7	65.5		0
C17	53.5		0
C1	80.5		0
C15	102.2		0
C5	103.7		0
C6	157.4		0
C14	160.0		0
C9	166.0		0
C11	172.0		0
C16	172.4		0
C4	179.0		0
L1	134.5		0
LC2, LC6	131.2	5.36	2
LC3, LC5	122.7	6.55	2
LC4	120.2	6.68	1
C22	172.7		0
C33	174.5		0
C83	174.5		0
C133	177.4		0
C173	171.6		0
C72	172.1		0
C182	172.6		0

Table S2: Assignment of all non-exchangeable ^{1}H and all ^{13}C chemical shifts in βPhCbi

assignment	δ^{13}C (ppm)	δ^{1}H (ppm)	Nprot
C1A	19.0	1.12	3
C12B	25.5	0.93	3
C81	22.1	1.89/2.21	2
C17B	13.4	1.24	3
C82	27.7	2.31/ 2.43	2
C177	14.9	1.14	3
C2A	11.6	1.45	3
C12A	15.0	1.57	3
C71	37.9	1.77/2.62	2
C171	37.7	1.95/2.02	2
C7A	14.7	1.83	3
C21	37.4	1.95/2.02	2
C31	20.9	2.05/2.24	2
C131	21.1	1.96/2.28	2
C172	23.6	2.65	2
C181	30.5	2.53	2
C32	28.0	1.93/2.57	2
C132	27.3	2.03/2.26	2
C151	11.1	2.57	3
C18	34.2	2.95	1
C51	11.4	2.56	3
C175	41.5	3.18/3.23	2
C13	48.4	3.63	1
C19	71.0	3.76	1
C8	50.0	3.69	1
C3	50.9	4.25	1
C176	61.7	3.89	1

Table S2: Assignment of all non-exchangeable ^{1}H and all ^{13}C chemical shifts in βPhCbi

assignment	δ^{13}C (ppm)	δ^{1}H (ppm)	Nprot
C10	91.1	6.66	1
C2	41.7		0
C12	42.2		0
C7	45.2		0
C17	54.6		0
C1	81.8		0
C15	103.5		0
C5	104.5		0
C6	159.3		0
C14	172.1		0
C9	168.0		0
C11	172.0		0
C16	171.4		0
C4	172.0		0
L1	127.0		0
LC2, LC6	128.4	5.10	2
LC3, LC5	123.2	6.62	2
LC4	120.4	6.75	1
C22	172.1		0
C33	172.1		0
C83	173.8		0
C133	174.3		0
C173	171		0
C72	170.8		0
C182	174.2		0

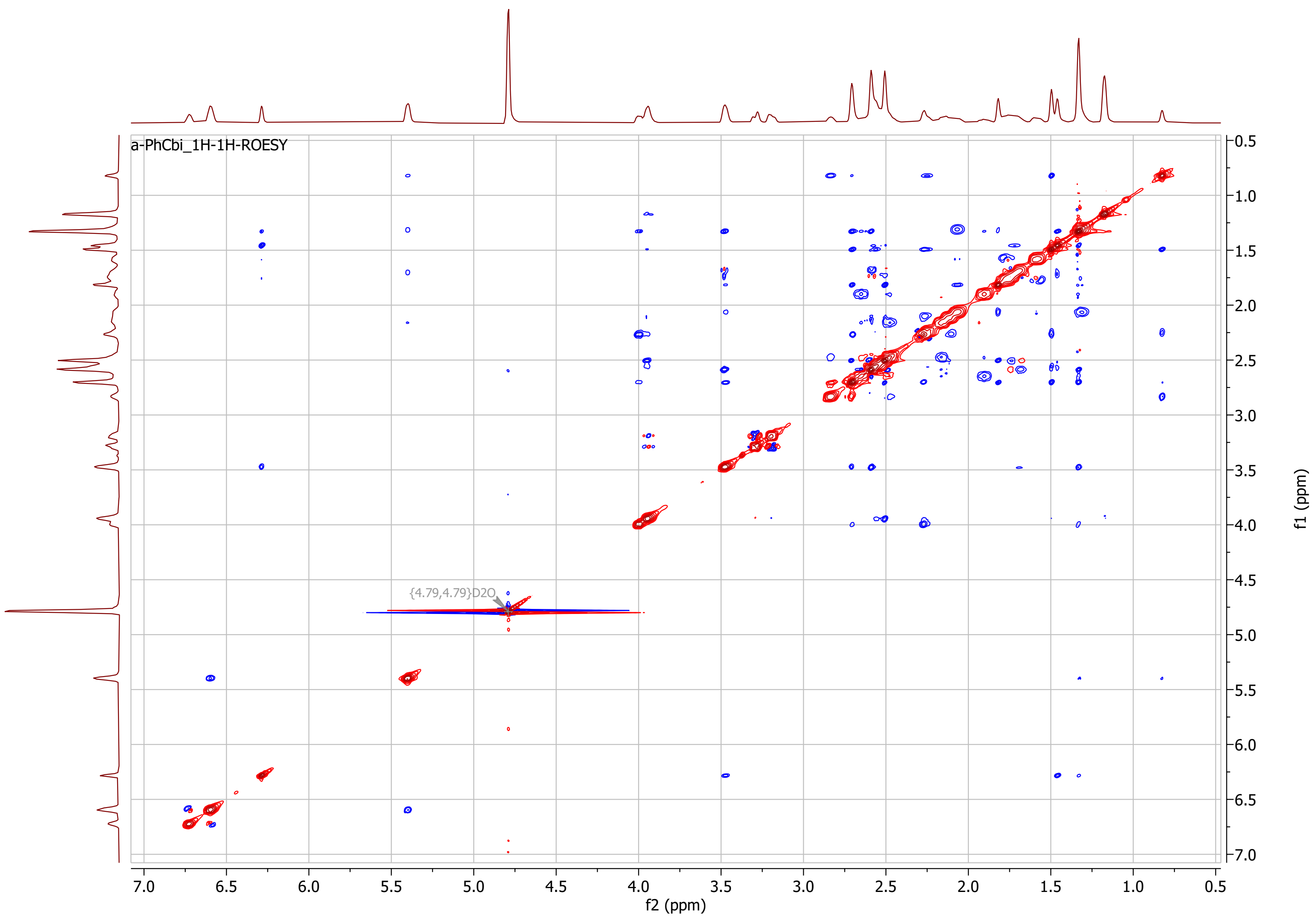

Figure S10: $^{1}H,^{1}H$-ROESY spectrum of αPhCbi (500 MHz NMR, D_2O, c = 8.5 mM, 298 K).

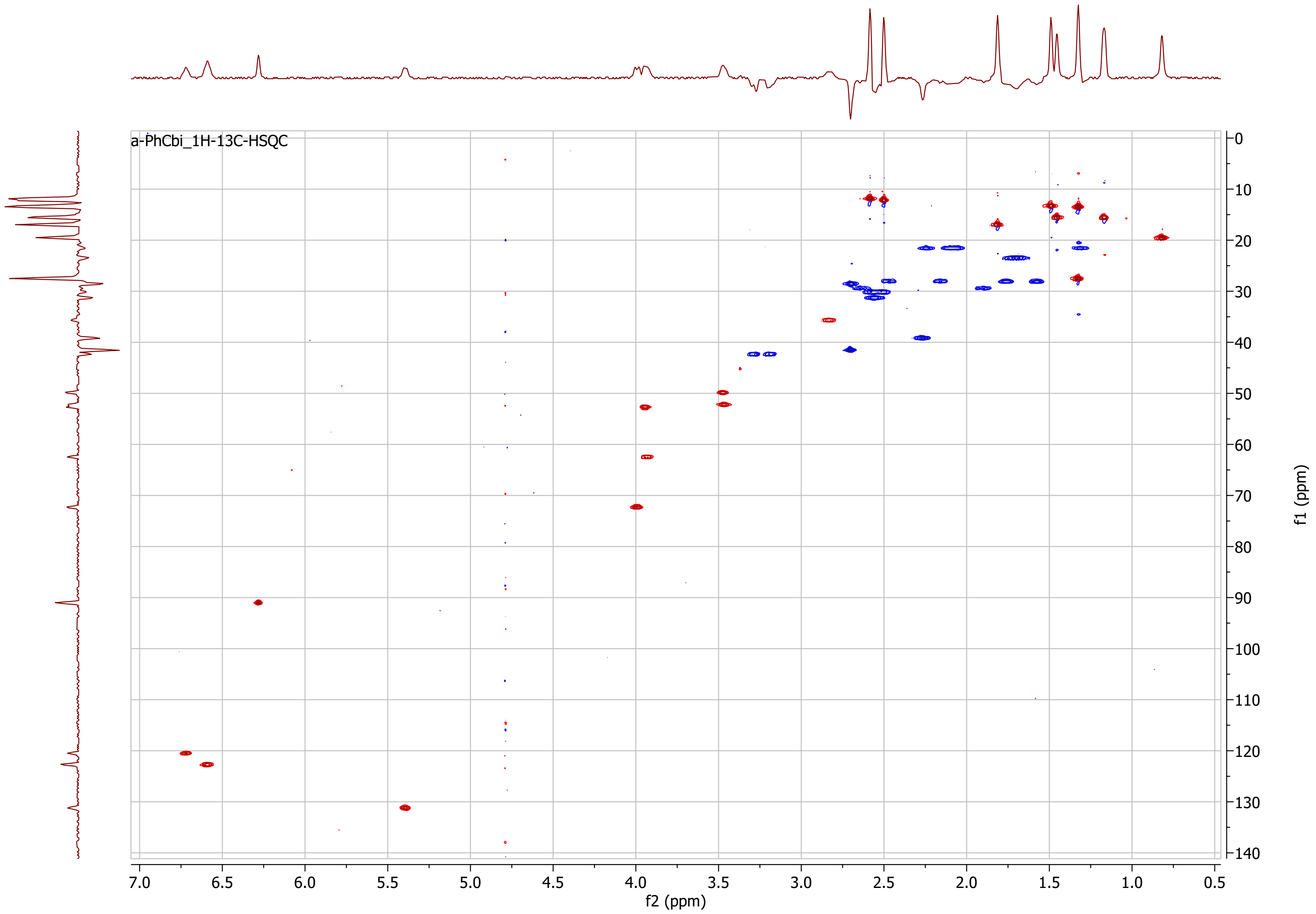

Figure S11: $^{1}H,^{13}C$-HSQC spectrum of αPhCbi (500 MHz NMR, D_2O, c = 8.5 mM, 298 K).

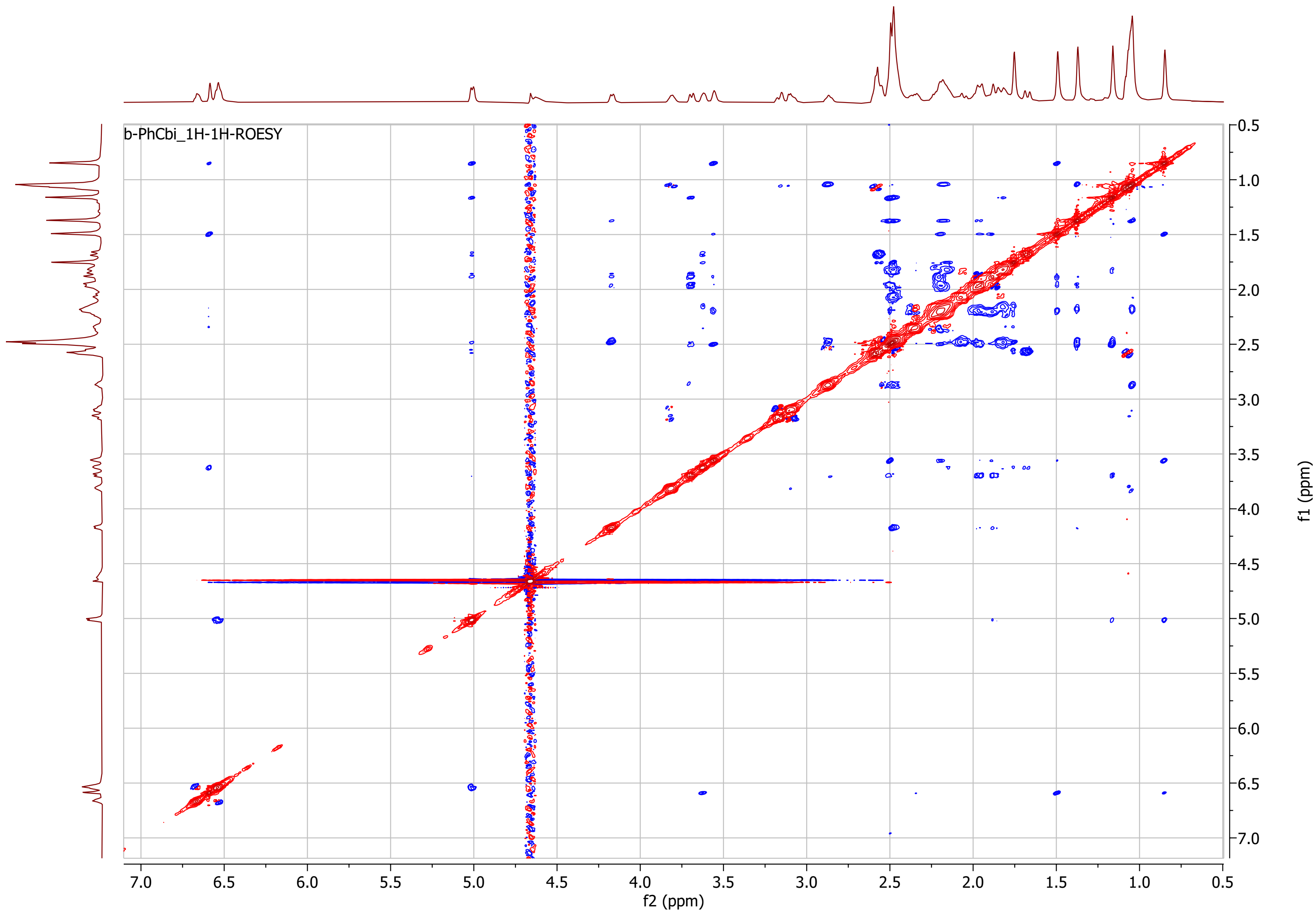

Figure S12: ^{1}H,^{1}H-ROESY spectrum of βPhCbi (500 MHz NMR, D_2O, c = 8.5 mM, 298 K).

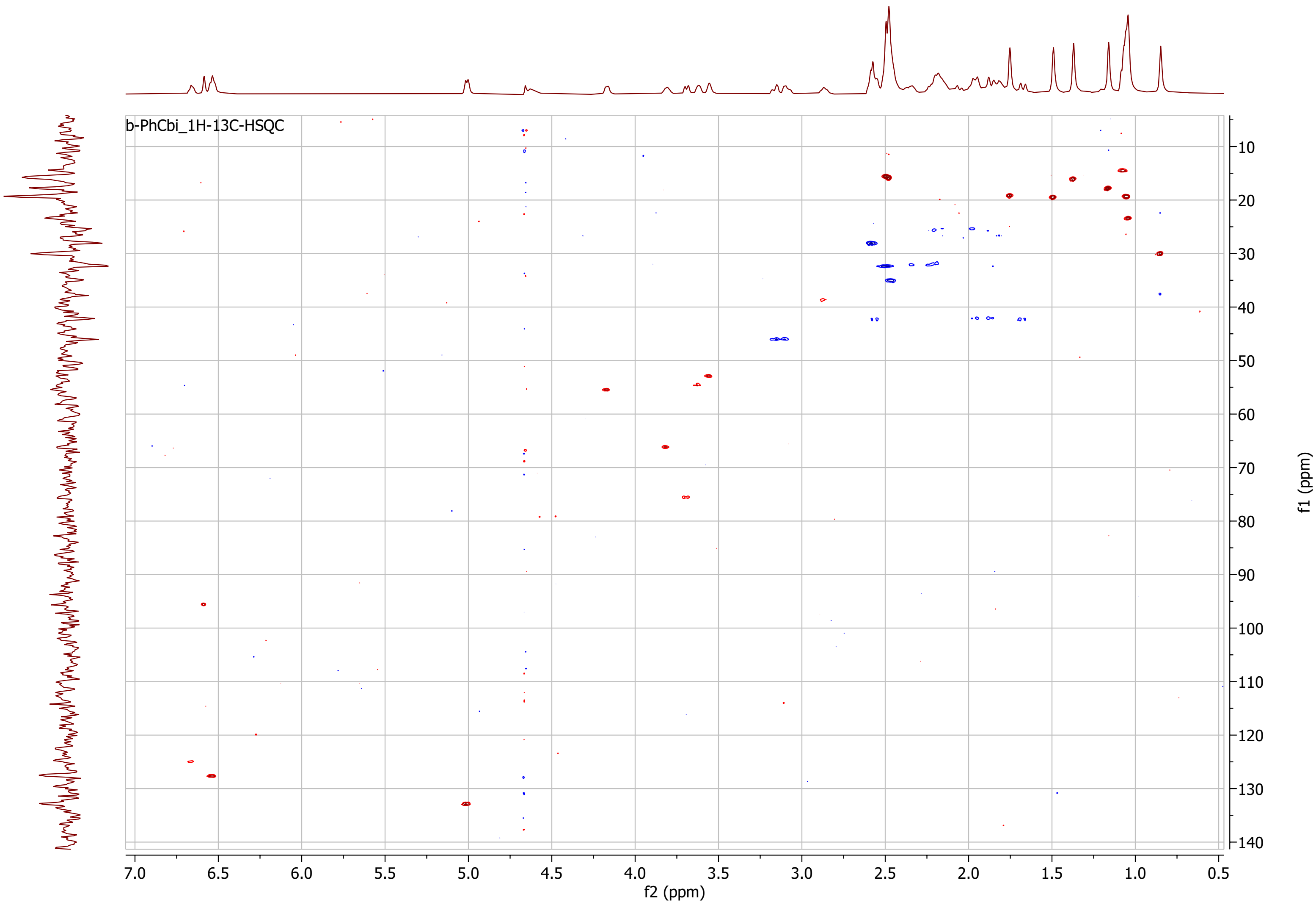

Figure S13: $^{1}H,^{13}C$-HSQC spectrum of βPhCbi (500 MHz NMR, D_2O, c = 8.5 mM, 298 K).

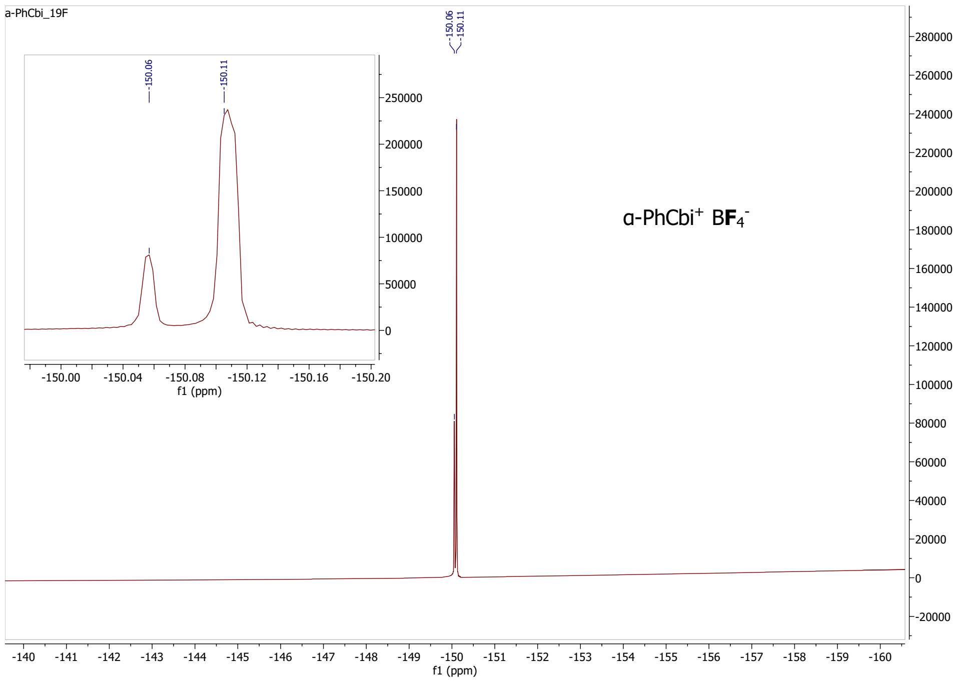

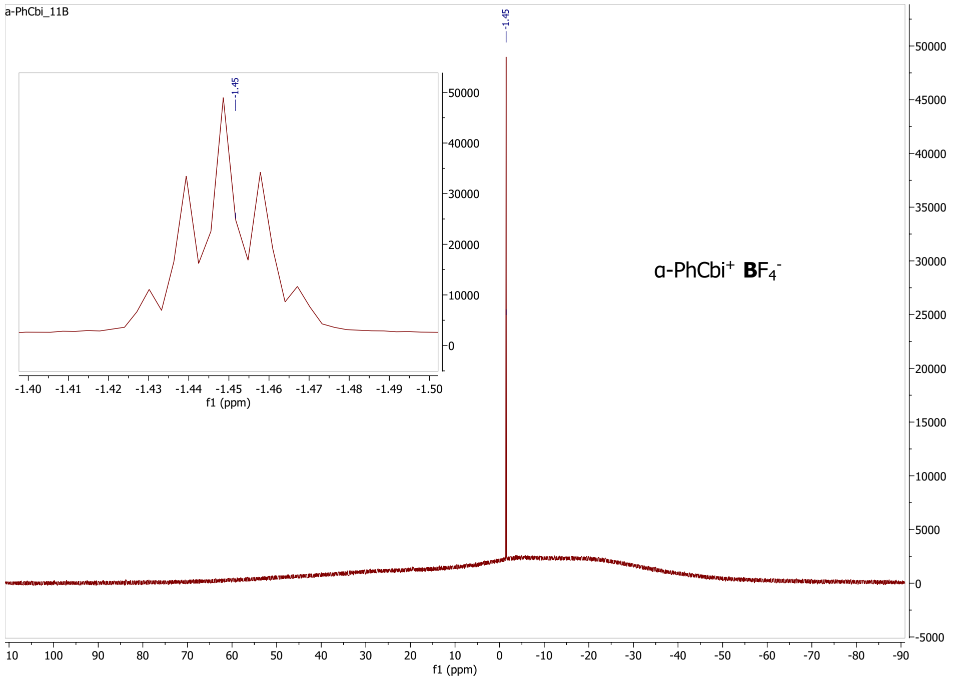

Figure S14: 128 MHz ^{11}B and 376 MHz ^{19}F NMR spectra of αPhCbi measured on 400 MHz Bruker Avance 4neo NMR spectrometer (D_2O, c = 1.84 mM, 298 K).

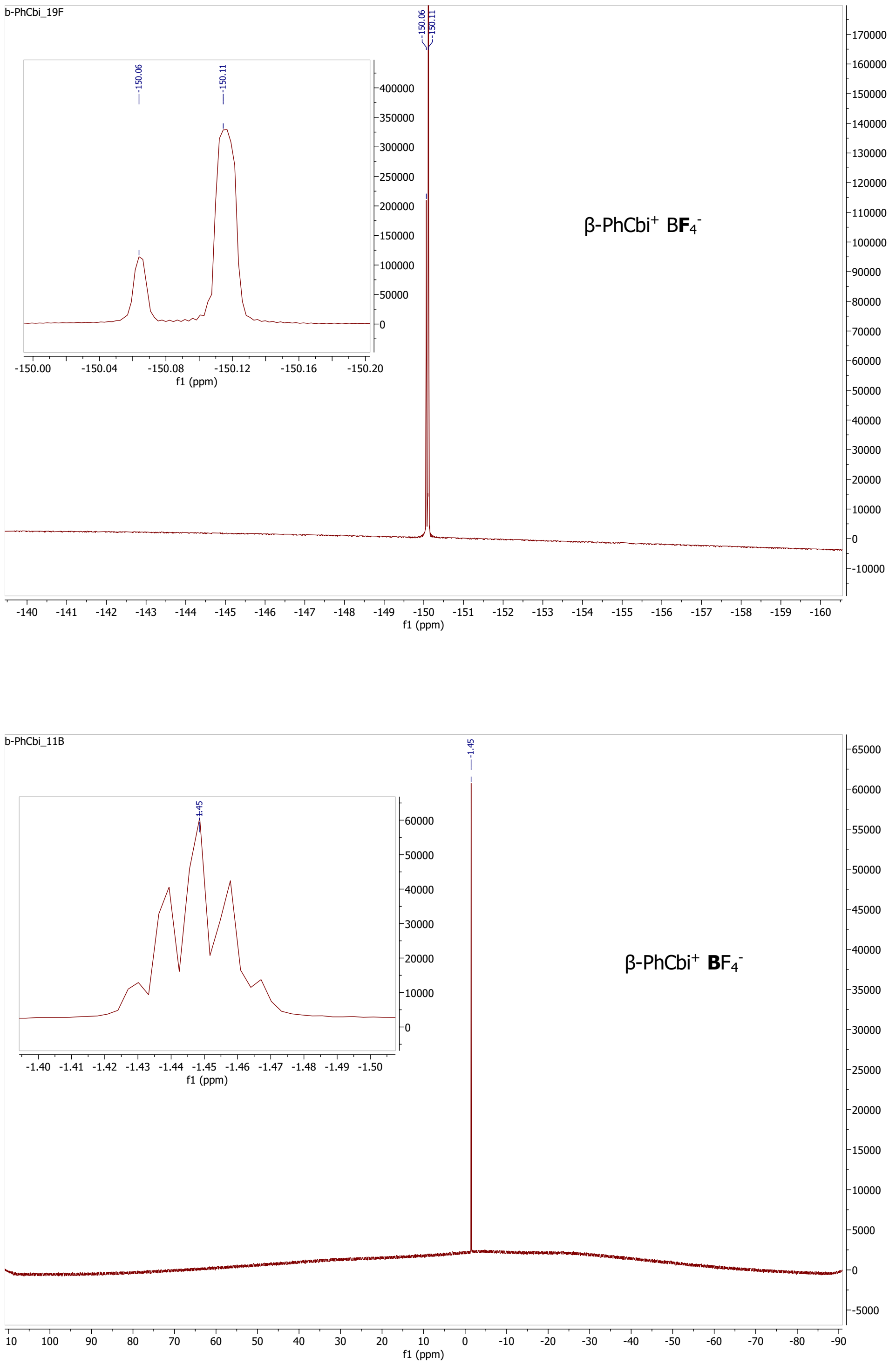

Figure S15: 128 MHz ^{11}B and 376 MHz ^{19}F NMR spectra of βPhCbi measured on 400 MHz Bruker Avance 4neo NMR spectrometer (D_2O, c = 1.84 mM, 298 K).

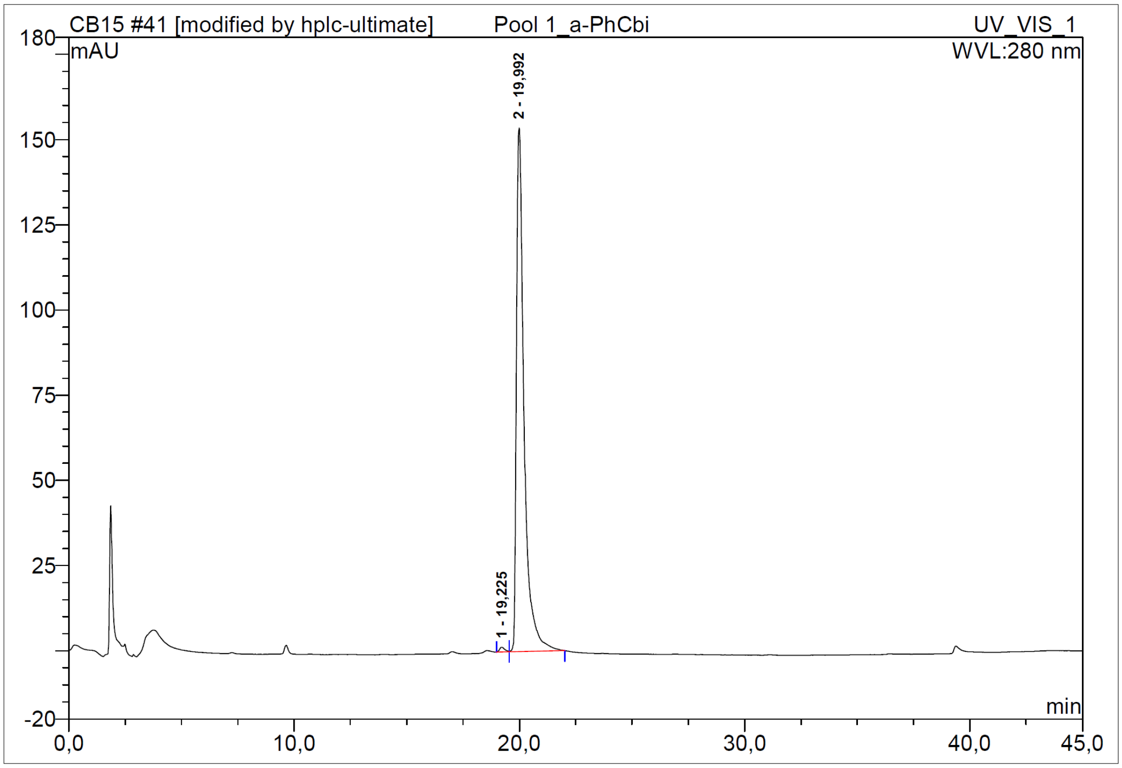

Figure S16: HPLC traces of αPhCbi.

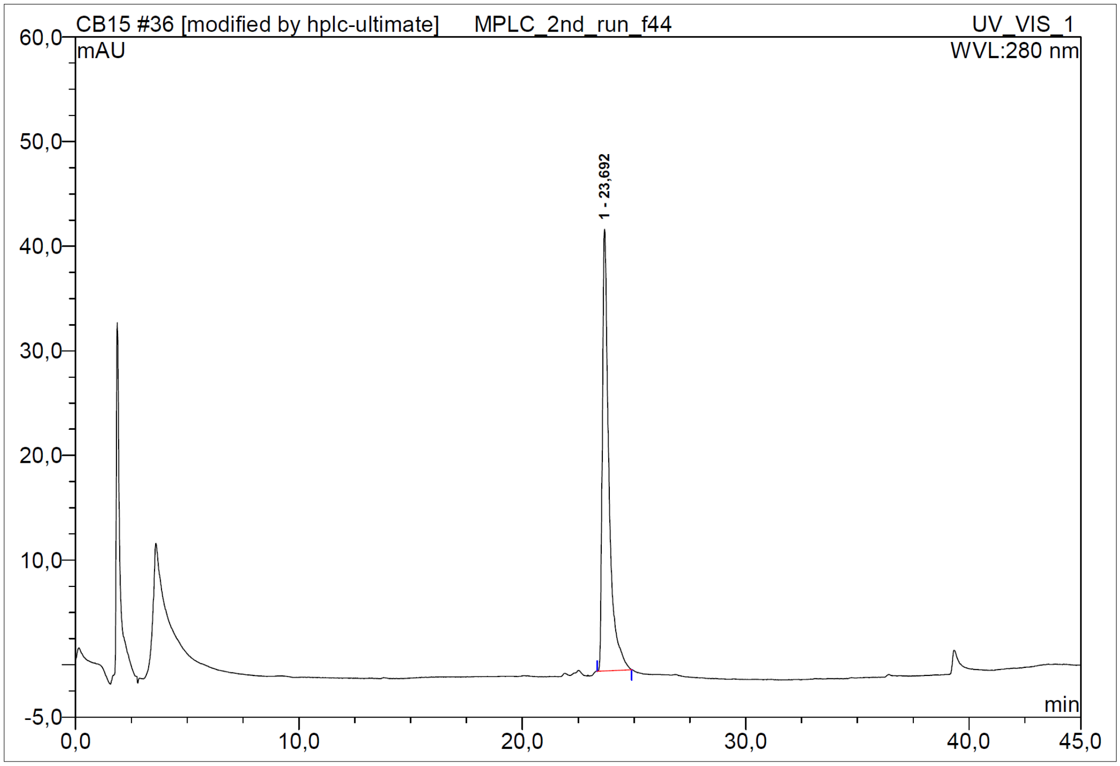

Figure S17: HPLC traces of βPhCbi.

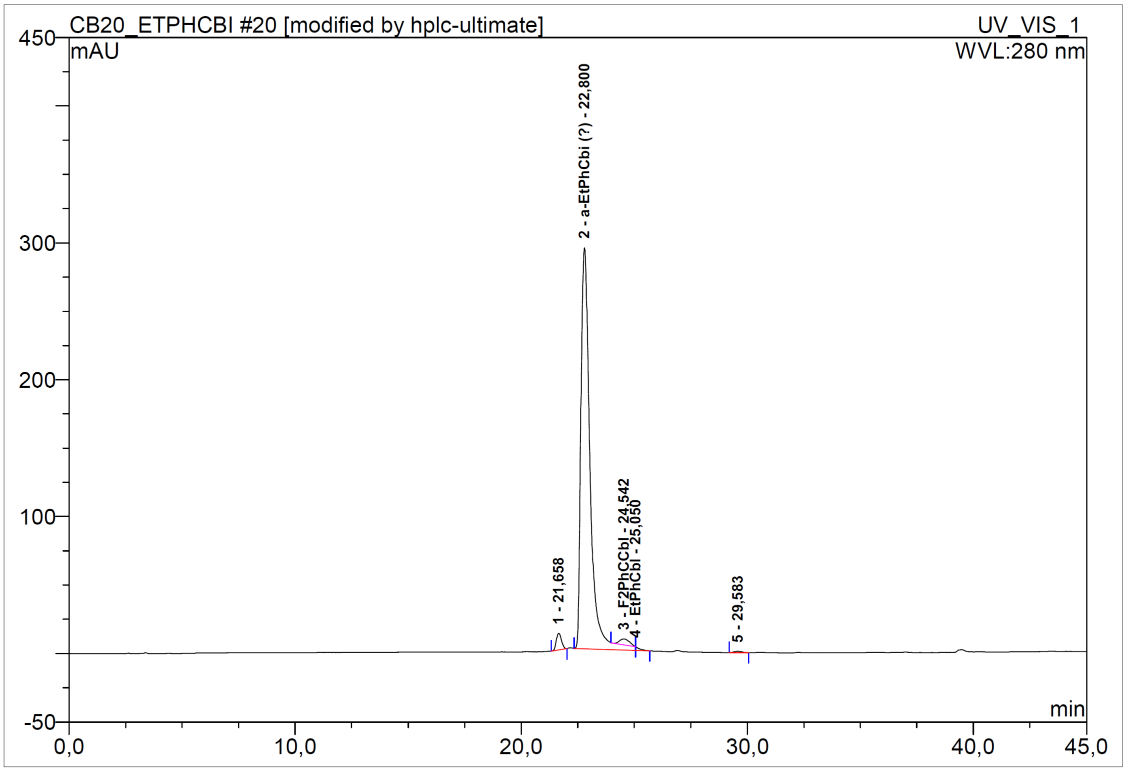

Figure S18: HPLC traces of αEtPhCbi.

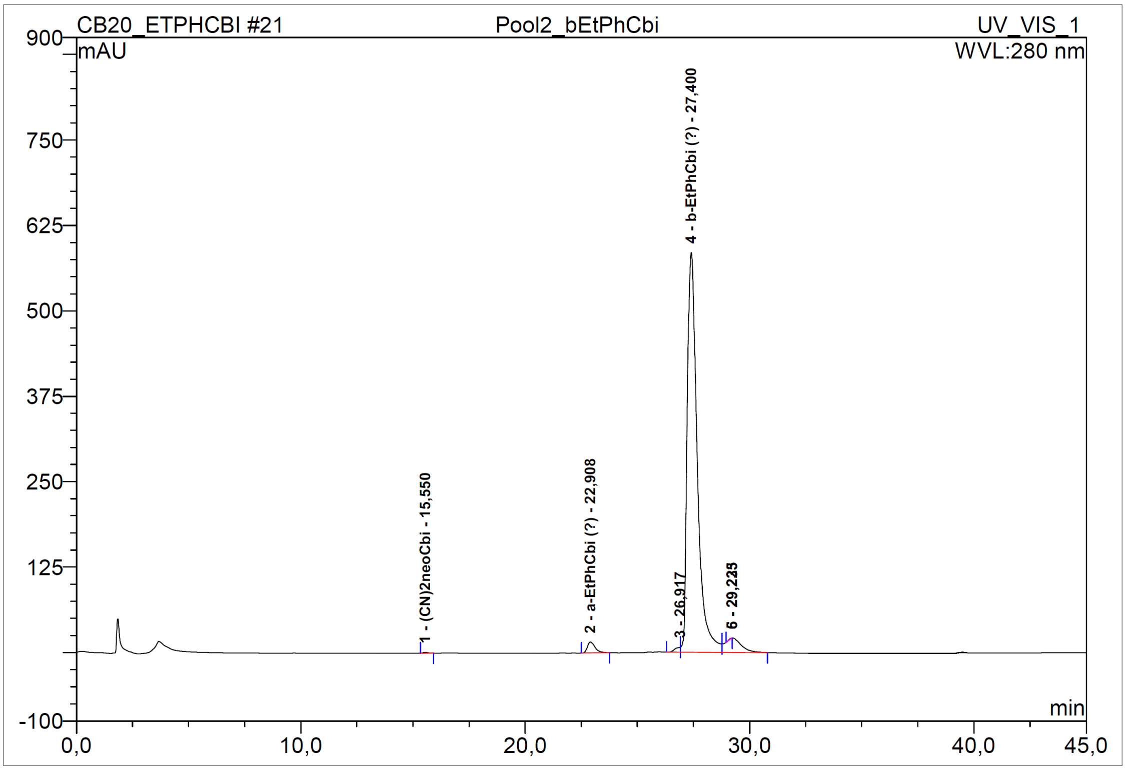

Figure S19: HPLC traces of βEtPhCbi.

T-CID measurements

In this section, for all the threshold-collision induced dissociation MS experiments carried out, the measured kinetic energy distributions, the ion intensity curves for CID, the cross-section curves and the L-CID fit results are provided in separate diagrams. In addition, the fitting results of all the L-CID fits are provided in a table for each compound.

T-CID data and L-CID fits of βEtPhCbi$^+$

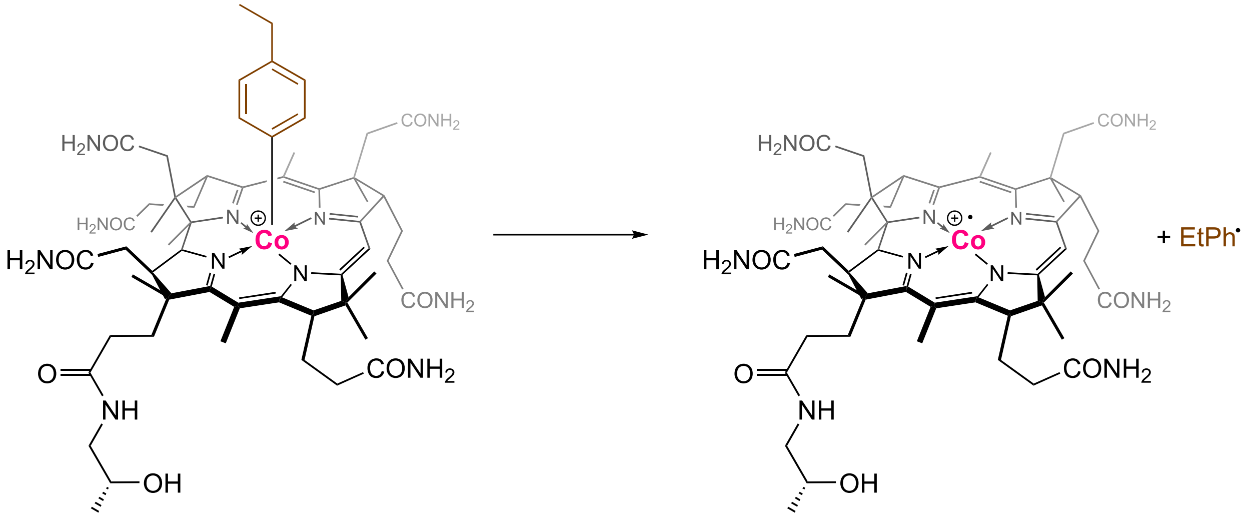

Figure S20: CID scheme for βEtPhCbi$^+$.

Table S3: L-CID fitting results.

#	E_0 / kcal mol^{-1}	ν_{eff} / cm^{-1}	α' / cm^{-1}	KED_{fwhm} / eV
1	40.68 ± 1.33	564 ± 39	6034 ± 424	
2	40.46 ± 0.78	353 ± 33	5778 ± 337	
3	40.08 ± 1.44	526 ± 20	5897 ± 479	
4	41.55 ± 0.16	539 ± 25	5710 ± 488	1.748
Avg.	40.6 ± 1.2	536 ± 29	5849 ± 435	–

Gas used: Xe, Number of rotors: 39, Degrees-of-freedom: 465

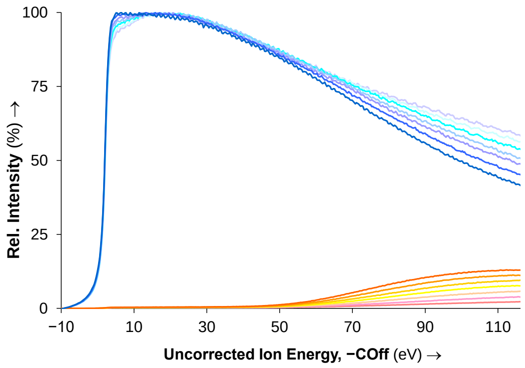

(a) Reactant and product intensities.

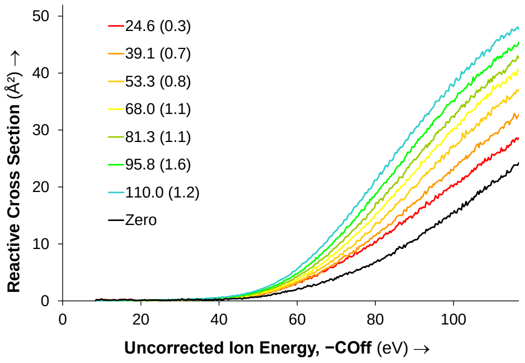

(b) Extrapolation to zero pressure.

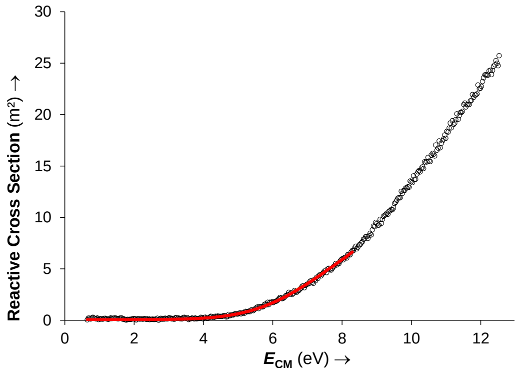

(c) L-CID fits of the σ_R.

Figure S21: T-CID plots. Measurement # 1.

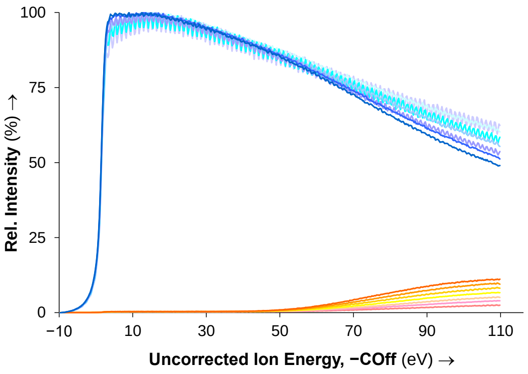

(a) Reactant and product intensities.

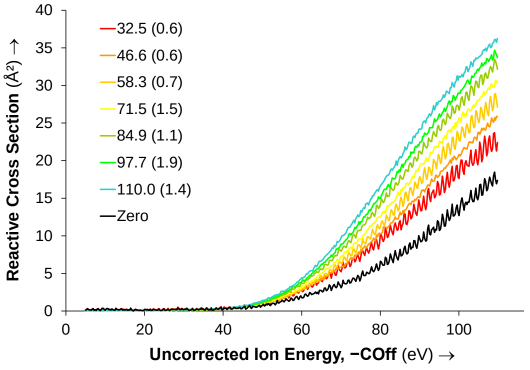

(b) Extrapolation to zero pressure.

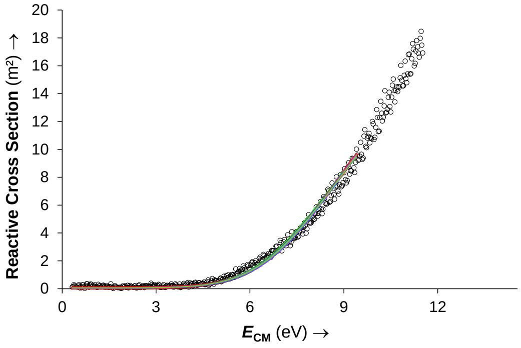

(c) L-CID fits of the σ_R.

Figure S22: T-CID plots. Measurement # 2.

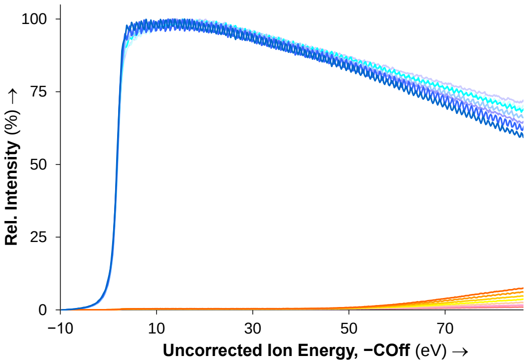

(a) Reactant and product intensities.

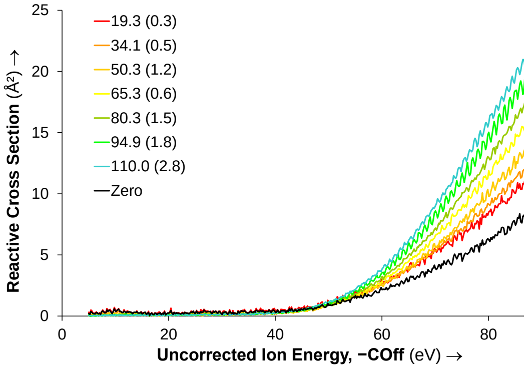

(b) Extrapolation to zero pressure.

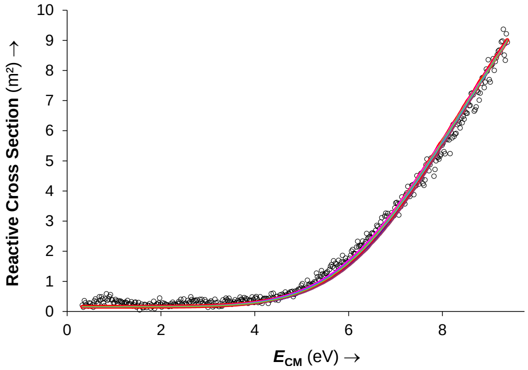

(c) L-CID fits of the σ_R.

Figure S23: T-CID plots. Measurement # 3.

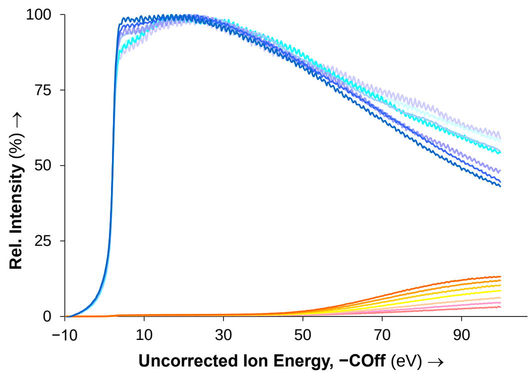

(a) Reactant and product intensities.

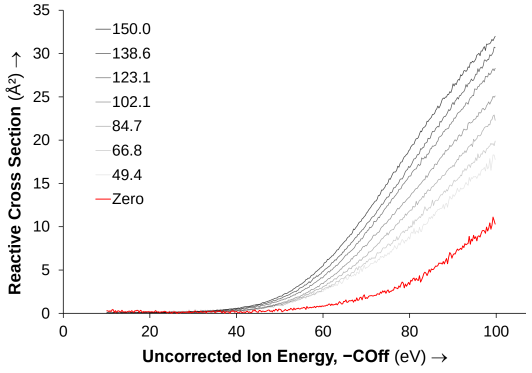

(b) Extrapolation to zero pressure.

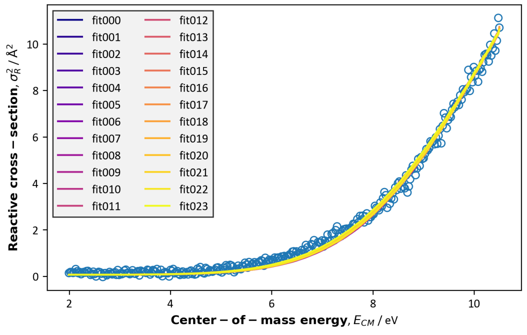

(c) L-CID fits of the σ_R.

Figure S24: T-CID plots. Measurement # 4.

T-CID data and L-CID fits of αEtPhCbi$^+$

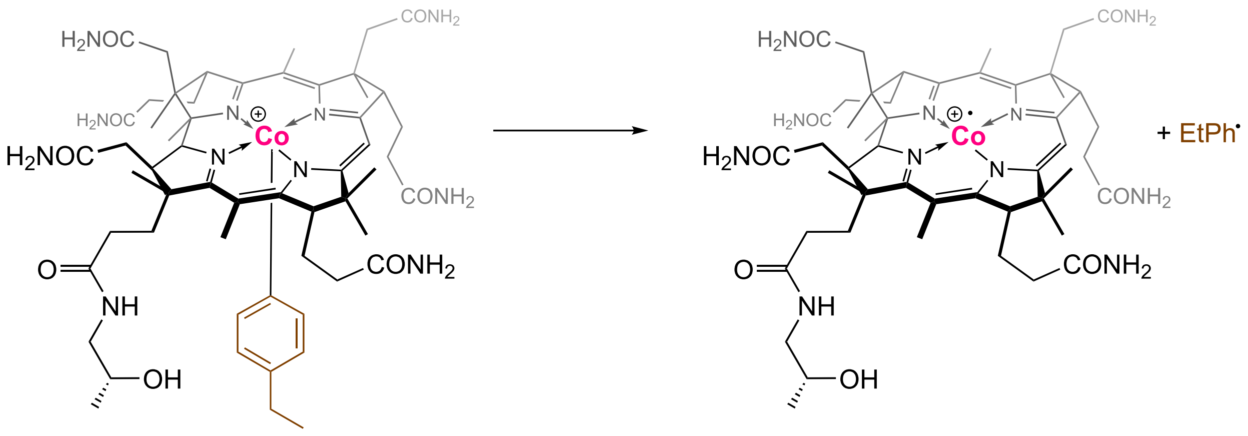

Figure S25: CID scheme for αEtPhCbi$^+$.

Table S4: L-CID fitting results.

#	E_0 / kcal mol^{-1}	ν_{eff} / cm^{-1}	α' / cm^{-1}	KED_{fwhm} / eV
1	43.8 ± 0.9	552 ± 41	6107 ± 283	1.7
2	46.2 ± 1.1	526 ± 19	5908 ± 382	1.82
3	45.4 ± 0.9	542 ± 26	6051 ± 331	1.7
Avg.	45.0 ± 1.4	534 ± 27	6043 ± 333	–

Gas used: Xe, Number of rotors: 39, Degrees-of-freedom: 465

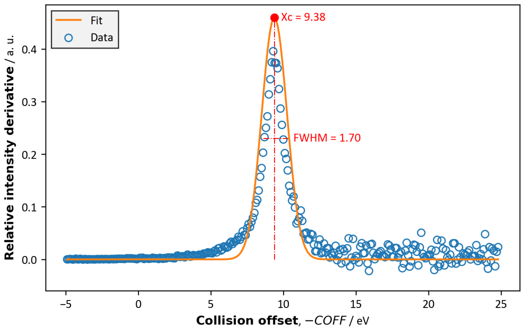

(a) KED.

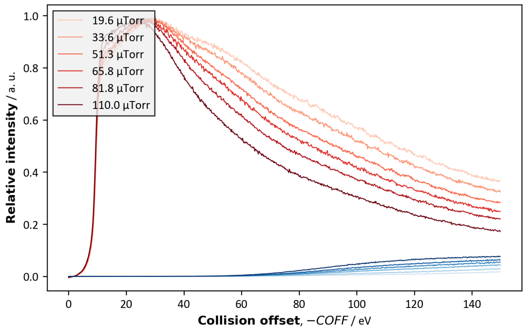

(b) Reactant and product intensities.

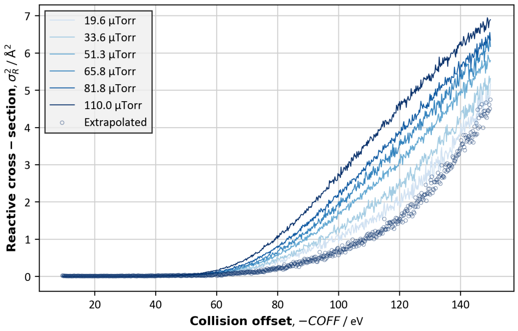

(c) Extrapolation to zero pressure.

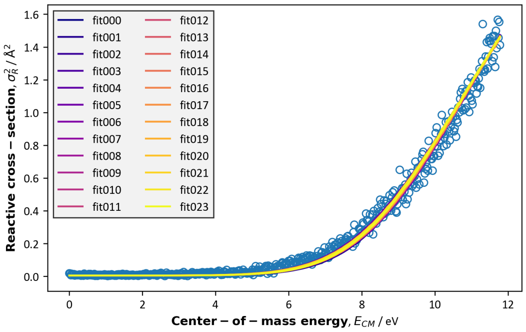

(d) L-CID fits of the σ_R.

Figure S26: T-CID plots. Measurement # 1.

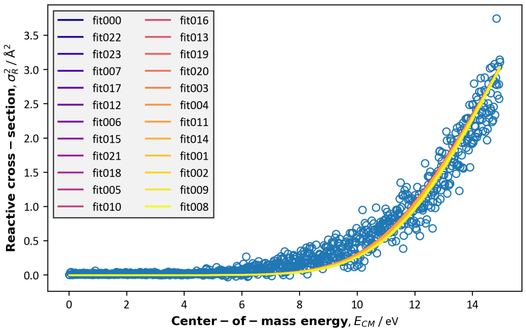

(a) KED.

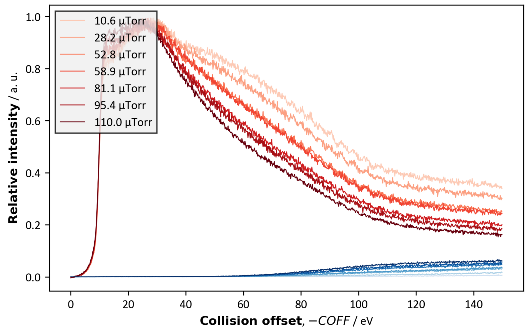

(b) Reactant and product intensities.

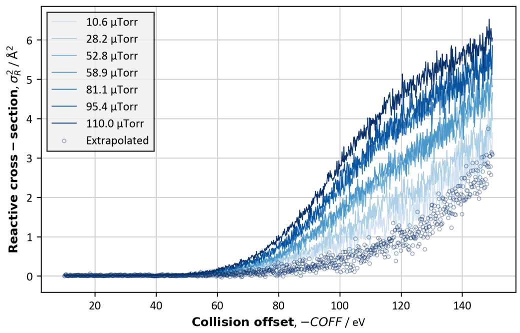

(c) Extrapolation to zero pressure.

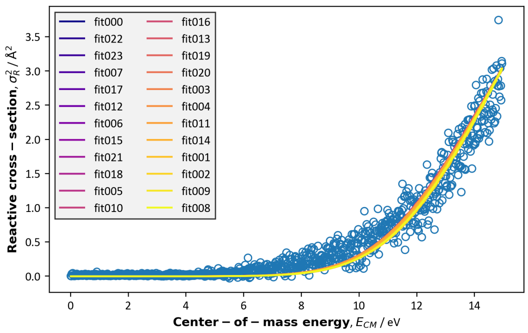

(d) L-CID fits of the σ_R.

Figure S27: T-CID plots. Measurement # 2.

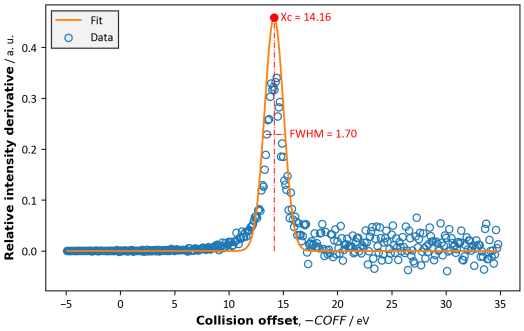

(a) KED.

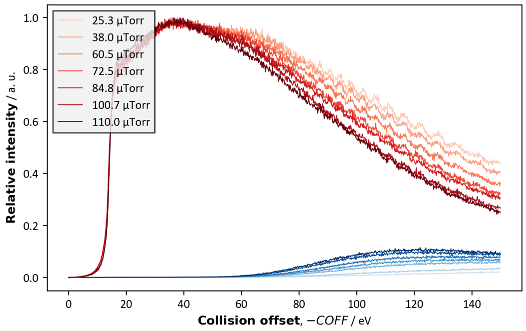

(b) Reactant and product intensities.

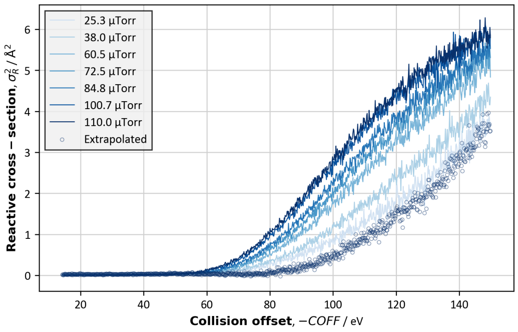

(c) Extrapolation to zero pressure.

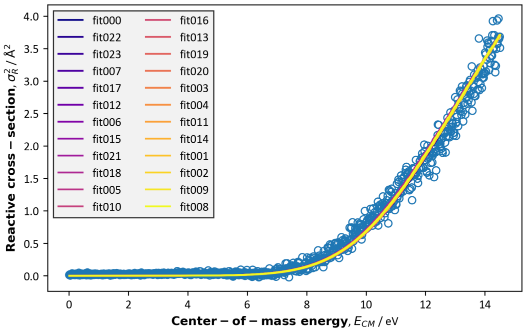

(d) L-CID fits of the σ_R.

Figure S28: T-CID plots. Measurement # 3.

T-CID data and L-CID fits of βPhCbi$^+$

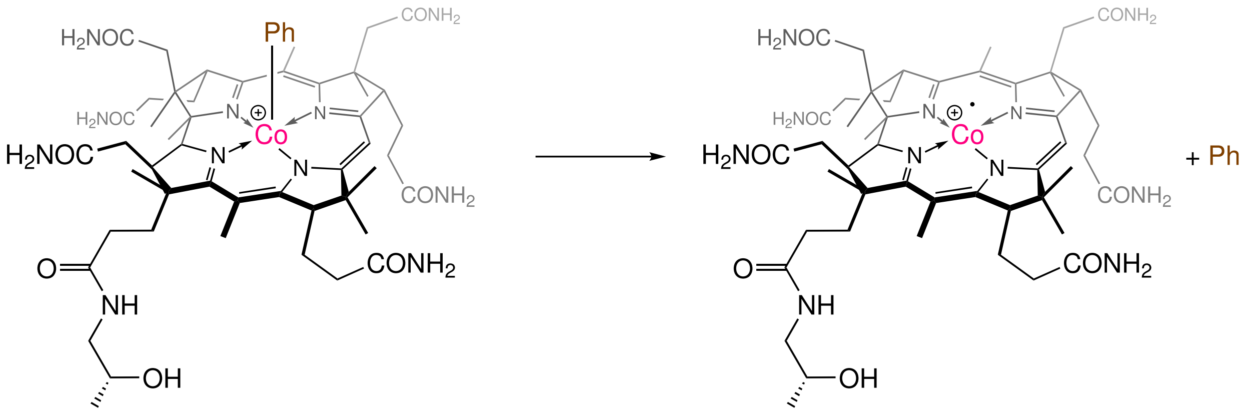

Figure S29: CID scheme for βPhCbi$^+$.

Table S5: L-CID fitting results.

#	E_0 / kcal mol^{-1}	ν_{eff} / cm^{-1}	α' / cm^{-1}	KED_{fwhm} / eV
1	41.72 ± 1.19	560 ± 51	5856 ± 401	1.23
2	39.2 ± 0.8	526 ± 17	5864 ± 277	2.08
3	38.5 ± 0.7	572 ± 41	5832 ± 304	2.43
Avg.	39.2 ± 1.4	535 ± 30	5951 ± 311	–

Gas used: Xe, Number of rotors: 38, Degrees-of-freedom: 447

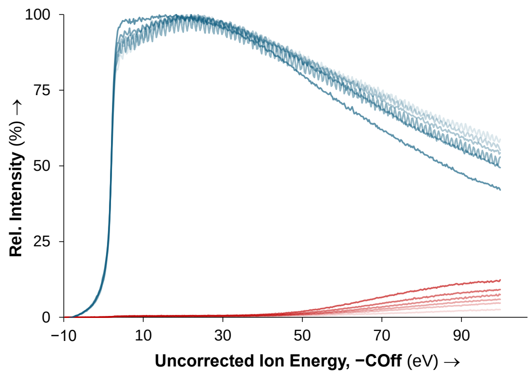

(a) Reactant and product intensities.

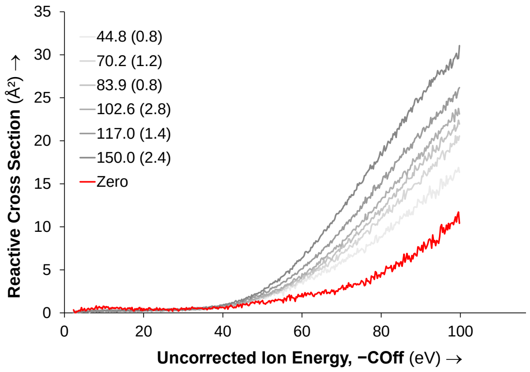

(b) Extrapolation to zero pressure.

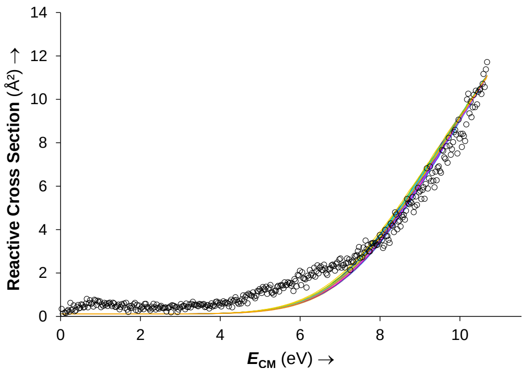

(c) L-CID fits of the σ_R.

Figure S30: T-CID plots. Measurement # 1.

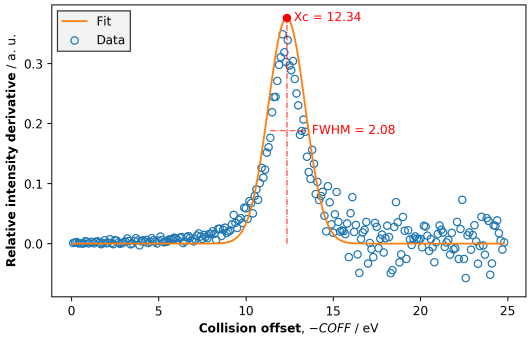

(a) KED.

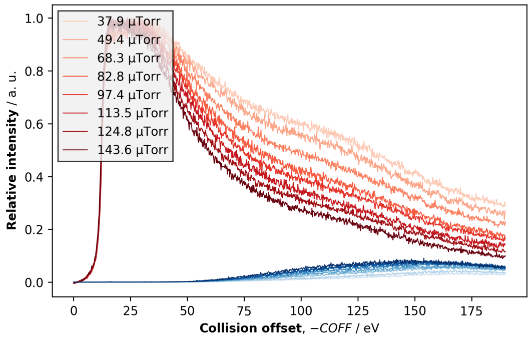

(b) Reactant and product intensities.

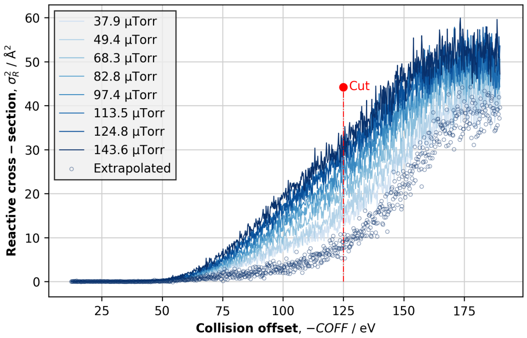

(c) Extrapolation to zero pressure.

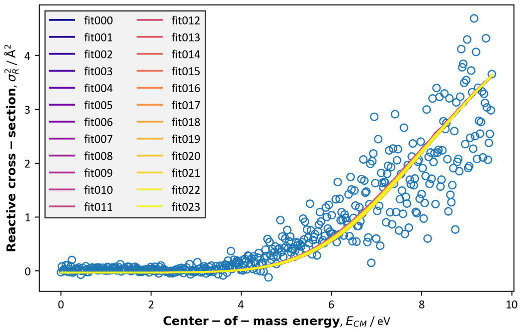

(d) L-CID fits of the σ_R.

Figure S31: T-CID plots. Measurement # 2.

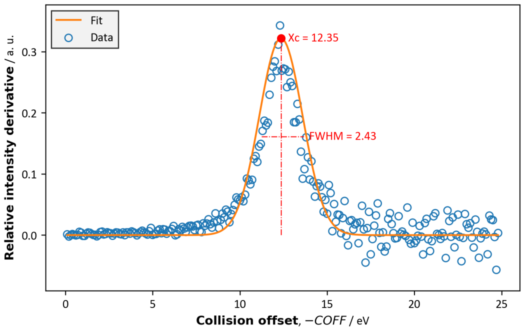

(a) KED.

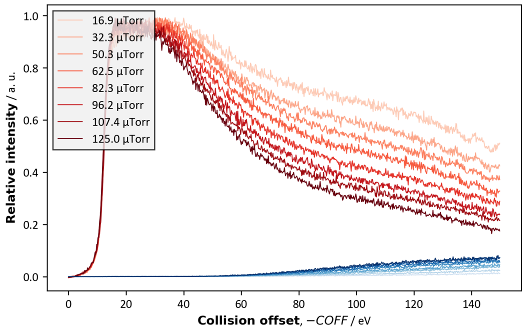

(b) Reactant and product intensities.

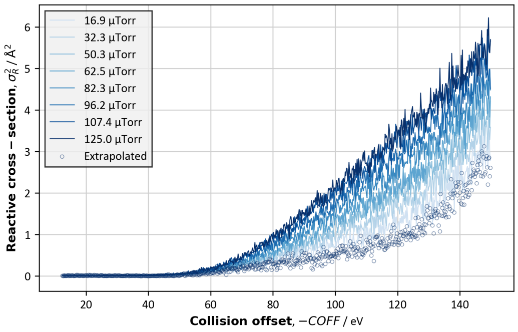

(c) Extrapolation to zero pressure.

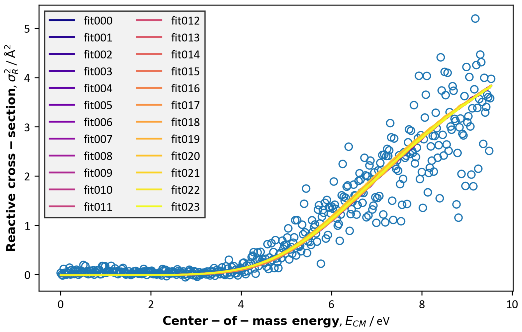

(d) L-CID fits of the σ_R.

Figure S32: T-CID plots. Measurement # 3.

T-CID data and L-CID fits of αPhCbi$^+$

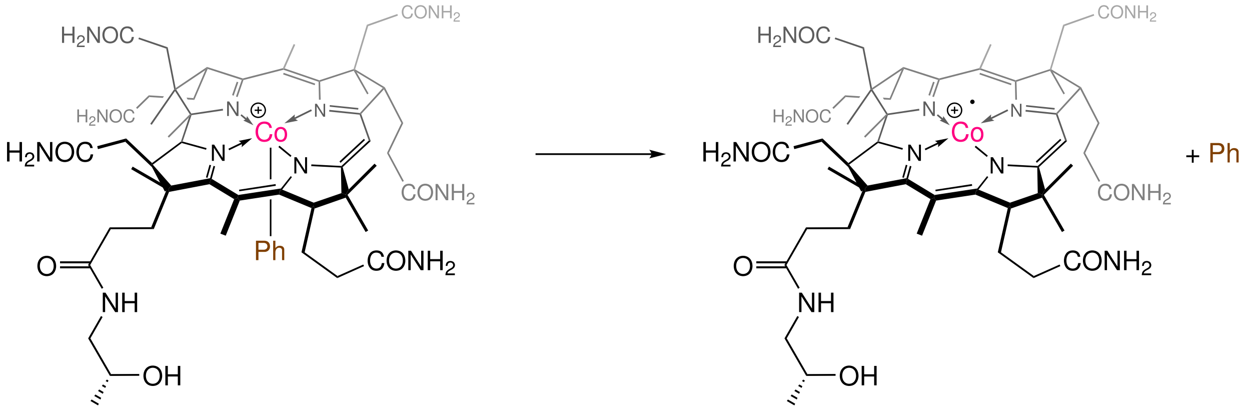

Figure S33: CID scheme for αPhCbi$^+$.

Table S6: L-CID fitting results.

#	E_0 / kcal mol^{-1}	ν_{eff} / cm^{-1}	α' / cm^{-1}	KED_{fwhm} / eV
1	46.7 ± 1.7	933 ± 71	5650 ± 480	1.17
2	46.4 ± 1.2	526 ± 17	5864 ± 277	1.54
3	46.4 ± 1.2	572 ± 41	5832 ± 304	1.89
Avg.	46.66 ± 0.14	610 ± 148	5902 ± 426	–

Gas used: Xe, Number of rotors: 38, Degrees-of-freedom: 447

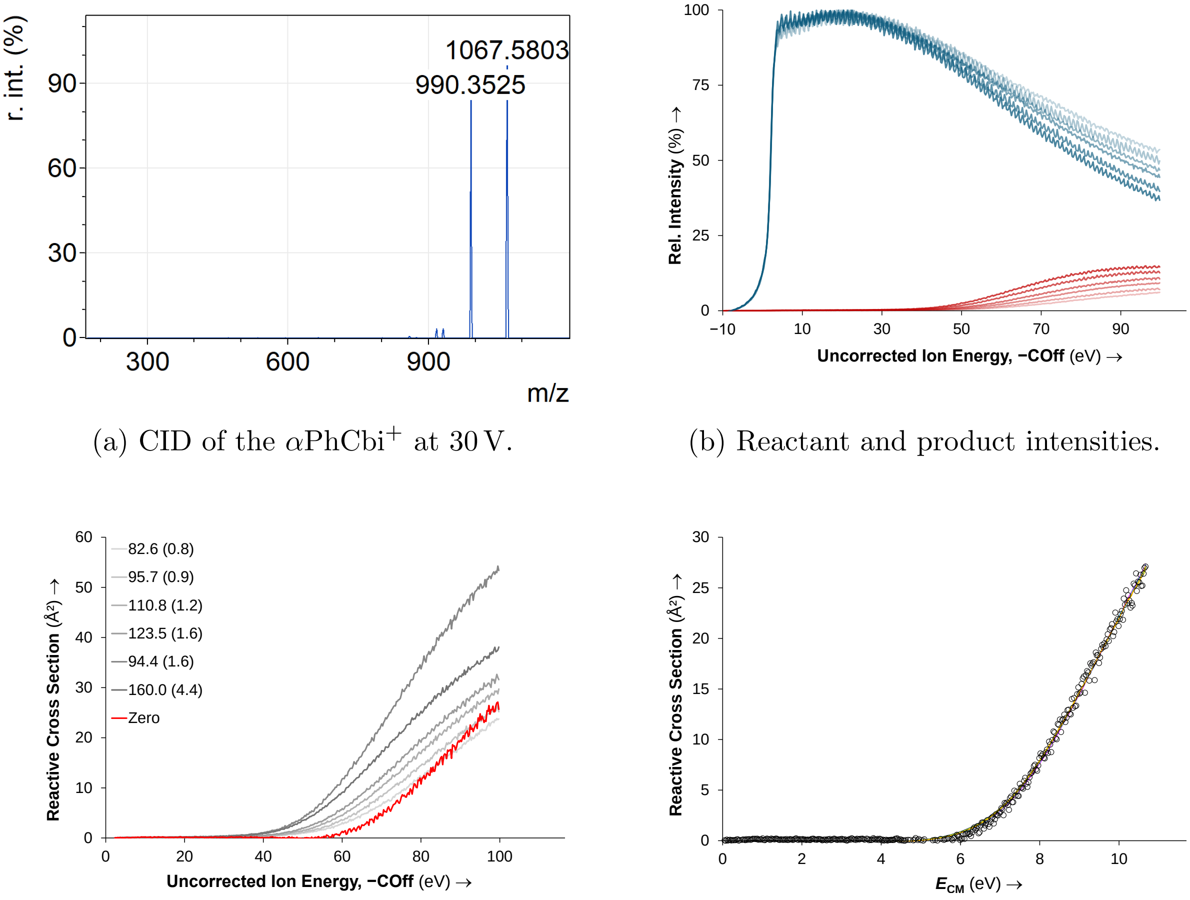

(a) CID of the αPhCbi$^+$ at 30 V.

(b) Reactant and product intensities.

(c) Extrapolation to zero pressure.

(d) L-CID fits of the σ_R.

Figure S34: T-CID plots. Measurement # 1.

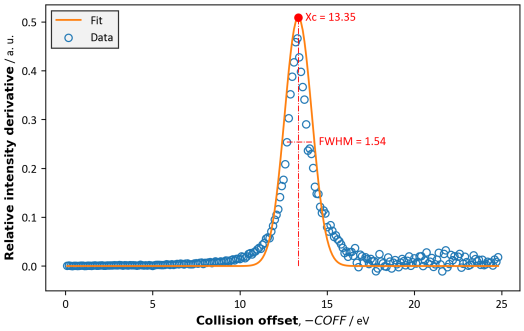

(a) KED.

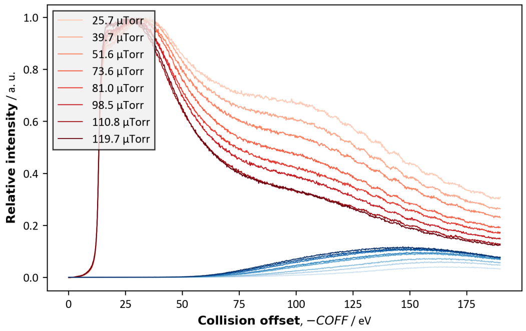

(b) Reactant and product intensities.

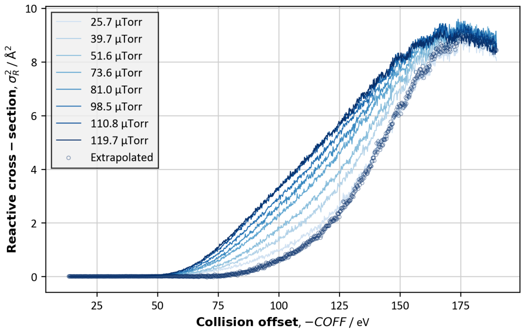

(c) Extrapolation to zero pressure.

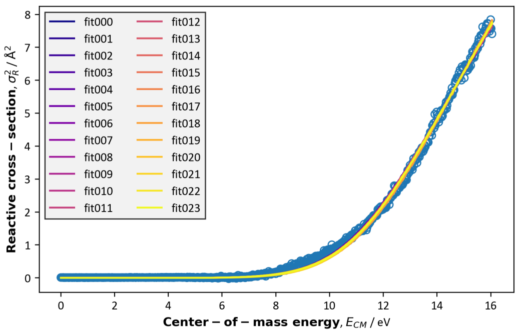

(d) L-CID fits of the σ_R.

Figure S35: T-CID plots. Measurement # 2.

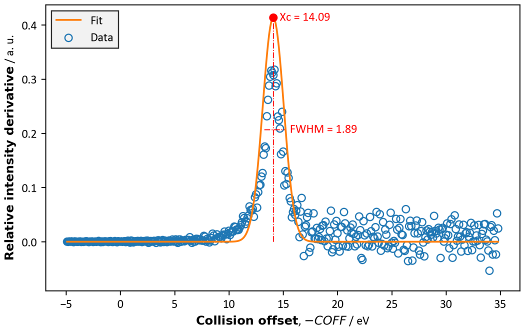

(a) CID of the αPhCbi$^+$ at 30 V.

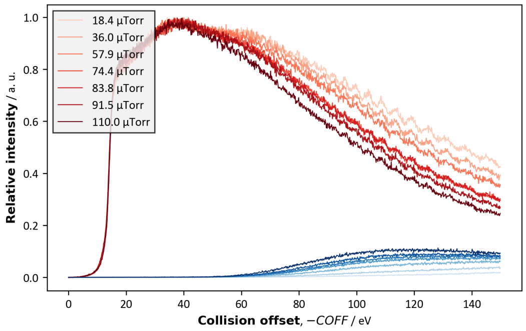

(b) Reactant and product intensities.

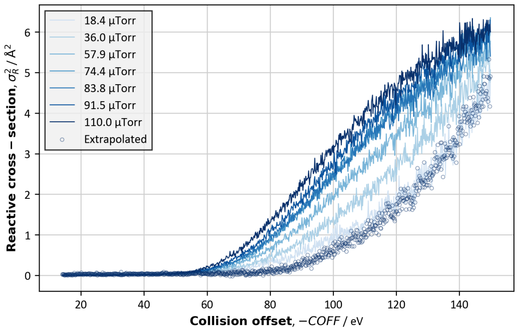

(c) Extrapolation to zero pressure.

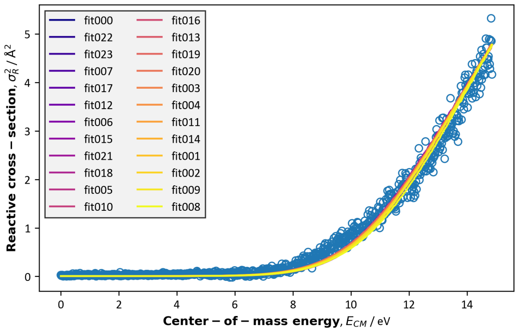

(d) L-CID fits of the σ_R.

Figure S36: T-CID plots. Measurement # 3.

References

(1) Butler, P. A.; Murtaza, S.; Kräutler, B. Partial Synthesis of Co α Co β-Dicyano-176-Norcobinamide. *Monatshefte für Chemie/Chemical Monthly* **2006**, *137*, 1579–1589.

(2) Bielawski, M.; Aili, D.; Olofsson, B. Regiospecific one-pot synthesis of diaryliodonium tetrafluoroborates from arylboronic acids and aryl iodides. *The Journal of organic chemistry* **2008**, *73*, 4602–4607.
